# High-utility conserved avian microsatellite markers enable parentage and population studies across a wide range of species

**DOI:** 10.1186/1471-2164-14-176

**Published:** 2013-03-15

**Authors:** Deborah A Dawson, Alexander D Ball, Lewis G Spurgin, David Martín-Gálvez, Ian R K Stewart, Gavin J Horsburgh, Jonathan Potter, Mercedes Molina-Morales, Anthony W J Bicknell, Stephanie A J Preston, Robert Ekblom, Jon Slate, Terry Burke

**Affiliations:** 1 Department of Animal and Plant Sciences, University of Sheffield, Sheffield, S10 2TN, UK; 2 School of Biological Sciences, University of East Anglia, Norwich, NR4 7TJ, UK; 3 Department of Biology, University of Delaware, Newark, DE, 19716, USA; 4 Current address: Department of Biology and Biochemistry, University of Bath, Bath, BA2 7AY, UK; 5 Current address: Estación Experimental de Zonas Áridas (CSIC), Almería, E-04120, Spain; 6 Current address: Departamento de Zoología, Universidad de Granada, Granada, E-18071, Spain; 7 Current address: Plymouth University, Marine Biology and Ecology Research Centre, Davy Building, Drake Circus, Plymouth, PL4 8AA, UK; 8 Current address: Department of Ecology and Genetics, Uppsala University, Norbyv. 18D, Uppsala, SE-75236, Sweden

## Abstract

**Background:**

Microsatellites are widely used for many genetic studies. In contrast to single nucleotide polymorphism (SNP) and genotyping-by-sequencing methods, they are readily typed in samples of low DNA quality/concentration (e.g. museum/non-invasive samples), and enable the quick, cheap identification of species, hybrids, clones and ploidy. Microsatellites also have the highest cross-species utility of all types of markers used for genotyping, but, despite this, when isolated from a single species, only a relatively small proportion will be of utility. Marker development of any type requires skill and time. The availability of sufficient “off-the-shelf” markers that are suitable for genotyping a wide range of species would not only save resources but also uniquely enable new comparisons of diversity among taxa at the same set of loci. No other marker types are capable of enabling this. We therefore developed a set of avian microsatellite markers with enhanced cross-species utility.

**Results:**

We selected highly-conserved sequences with a high number of repeat units in *both* of two genetically distant species. Twenty-four primer sets were designed from homologous sequences that possessed at least eight repeat units in both the zebra finch (*Taeniopygia guttata*) and chicken (*Gallus gallus*). Each primer sequence was a complete match to zebra finch and, after accounting for degenerate bases, at least 86% similar to chicken. We assessed primer-set utility by genotyping individuals belonging to eight passerine and four non-passerine species. The majority of the new *Conserved Avian Microsatellite* (CAM) markers amplified in all 12 species tested (on average, 94% in passerines and 95% in non-passerines). This new marker set is of especially high utility in passerines, with a mean 68% of loci polymorphic per species, compared with 42% in non-passerine species.

**Conclusions:**

When combined with previously described conserved loci, this new set of conserved markers will not only reduce the necessity and expense of microsatellite isolation for a wide range of genetic studies, including avian parentage and population analyses, but will also now enable comparisons of genetic diversity among different species (and populations) at the same set of loci, with no or reduced bias. Finally, the approach used here can be applied to other taxa in which appropriate genome sequences are available.

## Background

Microsatellite loci are suitable for a wide range of applications and have remained the most commonly used marker for studies of population structure and paternity since the early 1990s [1-3]. The use of microsatellites is likely to continue to be used for many years to come. They are comparatively cheap to genotype and provide more population genetic information per marker than biallelic markers such as single nucleotide polymorphisms (SNPs; [4]). A single set of microsatellite markers can be used to genotype several related species, but SNP markers lack cross-species utility, and are therefore only suitable for population and paternity studies where the project involves just a single species. Microsatellites can be successfully used for genotyping samples of low DNA concentration or low-quality samples (such as museum and non-invasive samples, e.g. feather, hair and faecal samples), in contrast to, for example, SNPs and genotyping-by-sequencing methods. A relatively large amount of DNA (typically 250 ng per individual) is usually required for SNP-typing versus >1 ng for microsatellite-based genotyping. Microsatellites have a wide range of other applications, and for some of these they have been found to be more suitable than SNPs, e.g. in genetic stock identification ([5], cf. [6]). They are the most convenient marker to establish if an individual (plant, for example) is a clone of its parent. They enable investigation of ploidy in a species, which for many species remains unknown. Plants and insects can be haploid, diploid or tetraploid, etc. and in some cases, one sex may be haploid and the other diploid (e.g. some wasp species). Finally, microsatellites enable the rapid identification of cryptic species (e.g. [7]) and have been used successfully to identify species hybrids (e.g. [8,9]).

Unfortunately, like most markers, the isolation, development and validation of microsatellite markers can take time to complete and therefore prove costly. Due to their low abundance in birds compared to other taxa [10,11], enrichment protocols are routinely employed to isolate avian microsatellite loci. The enrichment and cloning of microsatellite sequences is a skilled task, and is, therefore, often out-sourced, to be performed at specialist research facilities or by commercial laboratories. The use of 454-pyrosequencing can increase the number of loci isolated (e.g. [12]) but this also has to be performed at a specialist facility and can therefore increase costs [13]. Several weeks are then usually required for the in-house stages of primer testing and validating markers.

Moreover, the development and selection of microsatellite markers using a single population from an individual species often results in ascertainment bias [14]. Thus, even when markers amplify in multiple species, they are often most polymorphic in the same population and/or species from which they have been isolated (e.g. [15-19]), preventing meaningful cross-species comparisons. Ideally, any marker type would be applicable to several species to enable cross-species comparisons and allow investigation of karyotype and genome evolution. The cross-species utility of microsatellites is higher than other types of markers. However, when microsatellites are developed in the traditional way, from a cloned single species, their utility is normally limited to closely-related taxa.

Since the early demonstrations of cross-species microsatellite amplification in birds (e.g. [20], attempts have been made to identify a useful number of primer sets of high utility in a wide range of avian species. A small number of such primer sets of high cross-species utility have been identified (e.g. [21]; see also the BIRDMARKER webpage http://www.shef.ac.uk/nbaf-s/databases/birdmarker, [22]). Unfortunately, loci that are polymorphic are often rendered useless for genetic studies due to deviation from Hardy–Weinberg equilibrium and high null allele frequencies [23]. However, Durrant *et al*. [24], demonstrated, by testing the 34 *TG* conserved microsatellite markers developed by Dawson *et al*. [21], that it is possible to identify at least 20 *validated* polymorphic loci in species of Passeridae or Fringillidae (classification based on Sibley & Monroe [25]), with the term “validated” indicating that each locus, when assessed in a single population of unrelated individuals, adhered to Hardy–Weinberg equilibrium and had an estimated null allele frequency lower than 10%. Between 12–40 of such validated markers are normally sufficient for parentage and population studies (e.g. [26-28]), although some analyses, such as heterozygosity–fitness correlations, may require larger numbers of loci [29,30]. A large number of zebra finch (*Taeniopygia guttata*) expressed sequence tag (EST) microsatellite loci have been identified as useful in the blue tit (*Cyanistes caeruleus*) and, due to the relatively large genetic distance between zebra finch and blue tit, these are expected to be of utility in multiple species of Paridae [31]. However, although sufficient conserved markers probably exist for paternity and population studies of most species of Paridae, Passeridae and Fringillidae, additional loci are required to combine with existing conserved markers and enable genetic studies and cross-species comparisons in the large majority of bird species (including over 5,000 passerines and 4,000 non-passerines, [25].

To identify highly conserved microsatellite loci in the avian genome, the ideal scenario would be to compare homologous sequences in the two most genetically distant avian species*.* The two most genetically distant bird groups are the ratites and non-ratites [32]. However, there are relatively few species of ratites (n = 57, [25], none of which have as yet had their genomes sequenced (as of 10th February 2013). In order to attempt to identify such highly-conserved microsatellite loci in the avian genome, Dawson *et al*. [21] previously compared homologous sequences in two very distantly related species, the zebra finch and chicken (*Gallus gallus*). The primer sequences of these loci were a complete match to both zebra finch and chicken and the marker names were therefore given the prefix “*TG*” representing the first letters of the binomial names of these two species ***T****aeniopygia guttata* and ***G****allus gallus*. The zebra finch and chicken are both non-ratites but belong to two distantly related groups of birds and have the highest recorded genetic distance for any two bird species based on DNA: DNA melting temperature (Δ T_m_) hybridisation distances (28.0, [33]. Both of these species have now had their whole genomes sequenced and assembled (see http://www.ensembl.org).

Dawson *et al*. [21] identified loci that amplified in all non-ratite bird species, a high proportion of which were polymorphic in most species tested. This earlier study utilised microsatellites mined from zebra finch EST sequences with very strong similarity to their chicken homologue, but where the repeat region in zebra finch was not necessarily present in its chicken homologue. The longest uninterrupted string of dinucleotide repeat units in the sequenced zebra finch and chicken alleles was low for most loci (zebra finch: *n* = 3–15, mean 8 repeats; chicken: *n* = 0–13, mean 6 repeats). For the markers developed in this way, the proportion of loci polymorphic in a species was inversely related to the genetic distance from the “source” species – the “source species” being regarded as zebra finch, the species that contained the most uninterrupted microsatellite repeat units. Passerine species were regarded as those with a genetic distance of 12.8 or less from zebra finch based on DNA: DNA melting temperature (Δ T_m_) hybridisation distances [25]. On average, 47% of those *TG* loci amplifying were polymorphic in passerines and 22% in non-passerines (zebra finch and chicken data excluded; [21]. The variability of a locus is related to the number of repeats it possesses [34]. The decrease in polymorphism with increasing genetic distance may have been due to a correlated reduction in the number of repeat units in the target species compared to the source species. In this new study, we have attempted to identify markers that are polymorphic in a larger range of species.

We followed the approach of Dawson *et al*. [21] by identifying highly similar homologous sequences in two distantly related species (zebra finch and chicken). However, here we (1) selected homologous sequences in which *both* species contained repeat motifs, (2) attempted to align sequences that contained more repeat units than in the earlier study (≥ 8, in *both* species) and (3) we searched the whole genome for conserved microsatellite loci (i.e. not just for microsatellites in EST sequences, as performed by Dawson *et al*. [21]). Microsatellites with more repeat units generally have higher mutation rates [35,36] and are therefore expected to be more variable. The use of the whole genome was expected to increase the number of useful loci identified due to the huge increase in the number of microsatellite sequences that were now available. It is unclear if the source origin of the sequence (i.e. anonymous genomic sequence versus EST) would be expected to have any influence on locus variability. There is evidence that there is no difference between the variability of microsatellite markers developed from non-EST and EST sequences but other studies suggest non-EST markers may be more variable than those from ESTs (cf. [37-39]). We developed a set of conserved markers for 24 loci using the stated criteria and assessed their utility across a wide range of avian species. Additionally, we compared the utility of the new marker set to that of the previously-developed conserved marker set [21].

## Methods

### Identification of microsatellite loci in the zebra finch and chicken genome

In order to identify microsatellite sequences we searched the contigs and supercontigs of the unassembled zebra finch genome (now assembled and published by [40]) and the assembled chicken genome version 2.1 [41], using a version of the SPUTNIK software modified by Cornell University (http://wheat.pw.usda.gov/ITMI/EST-SSR/LaRota/, [42]. We identified sequences containing any dinucleotide repeat regions (CA, GA, AT, GC or their complements) which had more than ten repeats and which were at least 90% pure (i.e. >18 bp long; Table 1). We extracted 200 bp of sequence flanking either side of the repeat region, or all of the available sequence if it was less than 200 bp.

**Table 1:** Identification of avian microsatellite sequences of high cross-species utility*

**Motif**		**ZF**		**CH**	**ZF-CH consensus sequences created**		**Primer sets designed**	
	n	%	n	%	n	%	n	%
AT/TA	3,586	56	2,700	41	16	38	4	17
CA/GT	2,329	36	2,711	41	22	52	16	67
GA/CT	543	8	1,169	18	4	10	4	17
GC/CG	0	0	1	<0.1	0	0	0	0
Total	6,458		6,581		42		24	

*possessing at least eight dinucleotide repeat units and based on a search of the zebra finch (ZF) and chicken (CH) genomes using the marker development criteria outlined in the Methods section.

### Identification of highly-conserved microsatellite loci

The length of the sequence compared against another affects the strength of the E-value obtained. The zebra finch sequences extracted and used for the BLAST sequence comparison to chicken were 421–487bp long (Table 2). We attempted to create a zebra finch–chicken consensus primer set for all zebra finch microsatellite sequences that exhibited an NCBI BLAST E-value of E-59 or better (lower) when compared to their chicken microsatellite homologue (Table 2). BLAST E-value scores were obtained using standalone blastN (version 2.2.8 of Blast for 32-bit Windows; [43]).

**Table 2:** **Sequence origins, homology and primer sequences of 24 Conserved Avian Microsatellite ( *CAM *) loci**

**Marker**	Sequence origins: ZF: zebra finch contig name & position CH: chicken chromosome & base pair location*	**ZF seq. length (bp) and similarity to CH (E-value)**	**Homology to ESTs or genes Ŧ**	**Primer sequence (5**^**′ **^**- 3**^**′**^**) and fluoro-label ¥**	**No. of degen. bases in primer pair**	**Primer seq. similarity to CH (%ID) (& number of bases mis-matching) Ψ**
CAM-01	ZF: Contig4.1379:6555-6992	437	Gene	[F] [HEX]AAAGGCCAAG**R**CCAGTATG	1	[F] 100
	CH: chr2:67828480-67828907	9E-147		[R] CTCTCATCCACCCTGTTAGC		[R] 100
CAM-02	ZF: Contig5.1371:163550-163981	431	None	[F] [6FAM]GAATTAAAGA**Y**AGCAGATGCAGG	1	[F] 100
	CH: chr7:22132454-22132893	1.1E-96		[R] AGCTGATGAAATGAGAATGCAG		[R] 100
CAM-03	ZF: Contig5.1597:35280-35767	487	None	[F] [HEX]ATTAGCATAGCTCAGCATTGCC	1	[F] 91 (2)
	CH: chr7:24391832-24392259	2.2E-70		[R] CGAGCATTCAA**M**CCTGTCATC		[R] 95 (1)
CAM-04	ZF: Contig8.649:3118-3539	421	None	[F] [6FAM]TACCTCTGGC**Y**AAGGAACTG	1	[F] 90 (2)
	CH: chr1:133721521-133721942	6E-133		[R] GCTCAGAACATCAATCACTGC		[R] 100
CAM-05	ZF: Contig12.77:11232-11665	433	EST & gene	[F] [6FAM]TTACACAGACTGCAAACCGC	1	[F] 100
	CH: chr1:47660443-47660868	2.4E-72		[R] CTGTT**K**CTCTAGTAATGAGATCCTG		[R] 92 (2)
CAM-06	ZF: Contig12.342:17413-17858	445	Gene	[F] [HEX]GTGATGGTCCAGGTCTTGC	0	[F] 100
	CH: chr1:52304006-52304445	9E-115		[R] CAAGAGGAACAGATGAGGGTC		[R] 100
CAM-07	ZF: Contig12.442:2629-3062	433	EST & gene	[F] [HEX]AAATGATGAG**R**TCTGGGTGAG	2	[F] 100
	CH: chr1:53412026-53412463	2E-113		[R] CCATTTCCAAG**W**GATTTGC		[R] 100
CAM-08	ZF: Contig13.893:13419-13850	431	EST & gene	[F] [6FAM]AGAA**R**AAGCCACCCTCACAG	1	[F] 100
	CH: chr10:516461-516890	5E-79		[R] CTCGTTTCCATTGGCGTTG		[R] 95 (1)
CAM-09	ZF: Contig15.537:32597-33018	421	None	[F] [HEX]AGA**Y**ACACAGCCACCCCAGAG	3	[F] 86 (3)
	CH: chr4:17039238-17039667	1.6E-79		[R] CAC**W**TGTATCCACA**Y**GCTGAC		[R] 90 (2)
CAM-10	ZF: Contig16.130:3866-4309	429	EST & gene	[F] [6FAM]TATCC**M**GAGAATGGGCATC	2	[F] 89 (2)
	CH: chr13:1070809-1071238	4.4E-67		[R] **K**GCTCTCATTGTCATGCTG		[R] 95 (1)
CAM-11	ZF: Contig17.242:5423-5868	445	EST & gene	[F] [HEX]TGGTACAGGGACAGCAAACC	1	[F] 100
(Z-linked)	CH: chrZ:7888318-7888739	1.7E-89		[R] AGATGCTG**R**GAGCGGATG		[R] 100
CAM-12	ZF: Contig23.425:77718-78157	439	None	[F] [6FAM]TGGCA**R**TAA**W**TCCAGAGATTACC	3	[F] 100
	CH: chr2:62785492-62785919	1E-95		[R] CTG**R**CATTTGTCTTAAGCGTG		[R] 95 (1)
CAM-13	ZF: Contig28.55:8348-8785	437	EST & gene	[F] [HEX]TCAAATACAGCAGCAGGCAG	0	[F] 100
	CH: chr6:28449965-28450408	4E-140		[R] TTCATTACCAAACAGCATCCAG		[R] 100
CAM-14	ZF: Contig32.413:24503-24950	447	Gene	[F] [6FAM]G**Y**AAGTGAAAGCTAAAGAAAGCC	1	[F] 100
	CH: chr9:5323789-5324214	2.3E-92		[R] GGCAGTTCCAGCCATTTAC		[R] 100
CAM-15	ZF: Contig49.62:16781-17206	425	Gene	[F] [6FAM]**S**GACGACTCCTTTATTTCCC	2	[F] 90 (2)
	CH: chr1:73032096-73032543	9E-105		[R] TTCTGACTTCC**Y**CAGGTAACAC		[R] 100
CAM-16	ZF: Contig50.513:25871-26302	431	Gene	[F] [HEX]AGCCTTGAT**M**TTGGGAAGAGC	2	[F] 90 (2)
	CH: chr17:4598995-4599424	1.1E-85		[R] ATCCATACTC**Y**GTGCAACCTG		[R] 100
CAM-17	ZF: Contig56.179:11880-12303	423	EST	[F] [6FAM]CGGGTTGTAATCAAGAAGATGC	0	[F] 100
	CH: chr3:10551236-10551663	5E-141		[R] CTGCGGAGCAATTAACGC		[R] 100
CAM-18	ZF: Contig61.97:37926-38358	432	EST & gene	[F] [HEX]TTAAGAAGTTTACACCCAGCG	0	[F] 100
	CH: chr3:31888225-31888655	1E-106		[R] GCTAAATAACAGAGCCAGGAAG		[R] 100
CAM-19	ZF: Contig69.248:5308-5739	431	EST & gene	[F] [6FAM]TCTTGGAGGCAGATA**R**GAAGTG	1	[F] 100
	CH: chr1:199733800-199734239	4E-119		[R] GAGCAAGCAAAGATCACAAGC		[R] 100
CAM-20	ZF: Contig70.196:1579-2012	433	EST & gene	[F] [HEX]TAACAGGCAGGAATGCAGG	0	[F] 100
	CH: chr24:2939427-2939862	9E-105		[R] TCAGCCAGTGTTGGAGGTC		[R] 100
CAM-21	ZF: Contig74.100:2226-2651	425	Gene	[F] [6FAM]TGGGAGAACATTATAGCGTGAG	1	[F] 100
	CH: chr2:2408229-2408652	1.1E-96		[R] TTGAAATG**R**GAACCACGGAC		[R] 95 (1)
CAM-22	ZF: Contig75.34:11916-12343	427	None	[F] [HEX]**R**AG**R**GCCACTTTCACTCCTG	3	[F] 90 (2)
	CH: chr18:6214289-6214714	1.2E-76		[R] ATGCTGTGACACT**K**GGAGGC		[R] 100
CAM-23	ZF: Contig83.70:49198-49633	435	EST & gene	[F] [6FAM]CTCCACTTAGCTTGTAAATGCAC	1	[F] 96 (1)
	CH: chr6:31243934-31244369	2E-142		[R] CCAAG**R**AGTGCCCTAGATGTC		[R] 100
CAM-24	ZF: Contig122.74:8163-8588	425	None	[F] [HEX]CCCACTTCAGTCTTCAGAGC	0	[F] 100
	CH: chr1:2092872-2093301	1.8E-59		[R] TGGAGTATTTGGGATTGGAG		[R] 100

*, the zebra finch sequences were isolated by a search of the unassembled contigs and super contigs of the zebra finch genome and the chicken sequences were isolated by a search of the assembled chicken genome (v2.1). The sequence of each locus is provided in Additional file 2.

bp, base pairs;

*ZF*, zebra finch *Taeniopygia guttata*;

*CH*, chicken *Gallus gallus*;

F, forward primer sequence;

R, reverse primer sequence

¥, The forward and reverse primer sequences match 100% to zebra finch and 86–100% to chicken *Gallus gallus* when the degenerate bases are accounted for. The degenerate bases used in the primer sequences shown in bold and underlined, R = A or G, Y = C or T, M = A or C, S = C or G, W = A or T, K = G or T;

Ψ, calculated by dividing the number of bases matching chicken (after accounting for the degenerate bases) by the total length of the primer sequence;

Ŧ, assessed for (a) similarity to sequences in the NCBI nucleotide EST and nr/nt databases identified using blastn (distant homologies) settings and (b) for similarity to protein coding regions in the CH & ZF assembled genomes which was identified by the presence of exons within 5 kb of the source sequence (searches performed 30/09/2011). Details of the sequence homologues found are provided in Additional file 6.

### Creation of a consensus hybrid sequence and primer design

Consensus zebra finch–chicken sequences were created by aligning homologous sequences using MEGA3 software [44] and replacing mismatching bases and gaps with the code “n” to represent an unknown base. We used the zebra finch–chicken consensus microsatellite sequences to design primer sets using PRIMER3 software [45]. The primer sequences were designed from the consensus zebra finch–chicken hybrid sequence including “n” at those base pair locations where the zebra finch and chicken bases did not match. When necessary, we altered the “General Primer Picking Conditions” and set the “Max #N’s” parameter (maximum number of unknown bases (N) allowable in any primer) to “1” or “2” so that degenerate bases (if needed) could be included in the primer sequence. Primers were selected to have a melting temperature between 57–63°C and the maximum allowable difference in the melting temperature between the forward and reverse primer was set as 1.0°C. However, it should be noted that the melting temperature assigned to an unknown “n” base by PRIMER3 is an average of all four bases and not the melting temperature of any actual base. The real melting temperature of primer sequences including degenerate bases will be different to that requested in the PRIMER3 selection criteria and also stated in the PRIMER3 output. The actual melting temperature will therefore be 0.88/2.18°C higher than that stated if the actual base at the location of the degenerate base was a G/C and 0.55/2.41°C lower if an A/T. We manually selected the primer-binding sites to be positioned in regions where the sequences were highly similar between zebra finch and chicken and attempted to include as few degenerate bases as possible, but most primers (encompassing 18 pairs) required the inclusion of degenerate bases. These degenerate bases were placed at the sites where a base mismatch occurred between the zebra finch and chicken sequence in an attempt to make the primer sequences amplify in multiple species. We used a maximum of two degenerate bases per primer and a maximum of three per primer pair (Table 2). With two degenerate bases per primer the difference in true melting temperatures versus those calculated by PRIMER3 ranges from a maximum of -4.82°C (n × 2 versus T × 2) to +4.36°C (n × 2 versus G × 2). The (multiple) different combinations of alternative primer sequences due to the inclusion of degenerate primer bases were not checked for adherence to PRIMER3 primer design criteria prior to ordering the primer sets due to the complexity of performing this task. The forward primer of each primer set was labelled with either a HEX or 6-FAM fluorescent dye (Table 2). The loci were named with the prefix *CAM* representing “**C**onserved **A**vian **M**icrosatellite”.

### Genome locations

**Figure 1:**
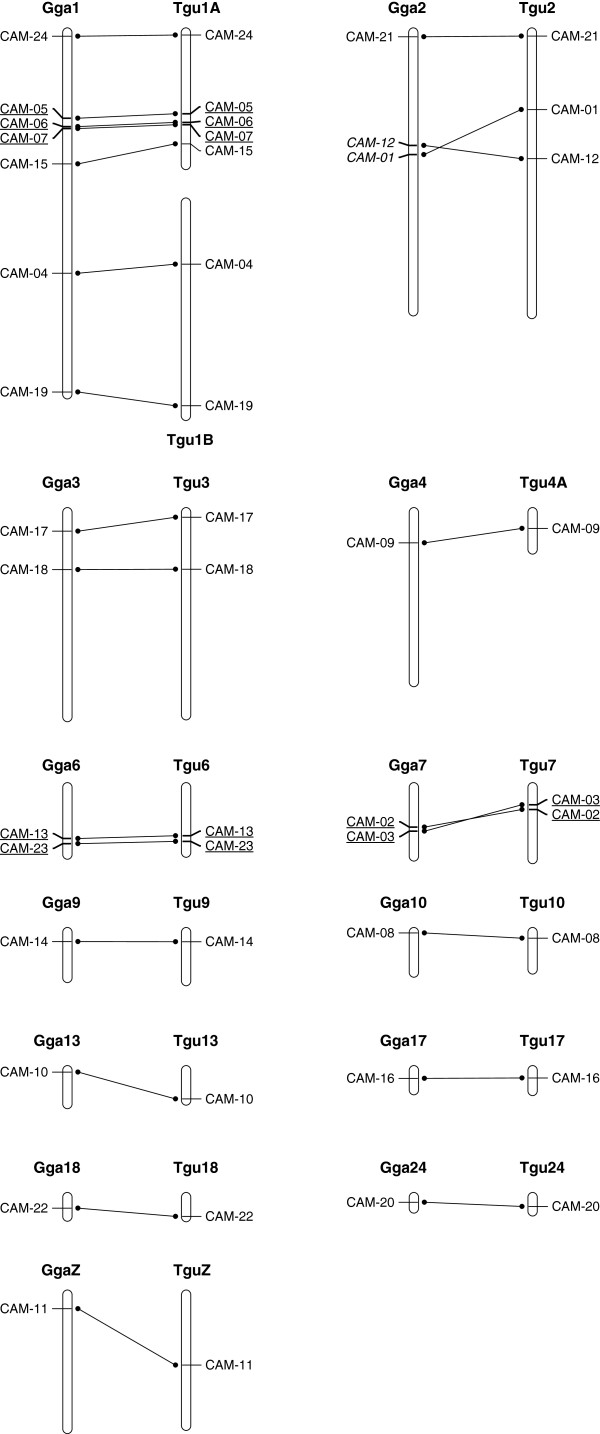
**Chromosome locations of the *CAM *loci in the chicken and zebra finch genomes.** Gga, chicken (*Gallus gallus*) chromosome. Tgu, zebra finch (*Taeniopygia guttata*) chromosome. The exact chromosomal locations of the loci (in base pairs) are provided in Table 2. Those loci underlined are less than 5Mb apart and may display linkage disequilibrium.

All of the sequences were assigned chromosome locations in the zebra finch and chicken genomes by performing a BLAT search against each genome, using the masked genome and the distant homologies settings implemented on the ENSEMBL webpage (http://www.ensembl.org/Multi/blastview; methods as in [46,47]; Table 3, Figure 1). The genome assemblies used were the Taeniopygia_guttata-3.2.4 (v 1.1), released 14 July 2008 [40] and the chicken genome assembly version 2.1 [41]. The locations of the loci were displayed using MAPCHART software [48].

**Table 3:** **Repeat motif, chromosome locations and locus variability of 24 Conserved Avian Microsatellite ( *CAM *) loci**

**Marker**	**Repeat motif type in ZF and CH β**	**Details of repeat motif in zebra finch and chicken β**	**Chr. location**	**Sp. typed**	***n***	**#A**	**Exp. length in ZF or CH (bp)^**	**Minimum expected allele size in ZF or CH (bp)^**	**Obs. allele size range in ZF or CH (bp)**
CAM-01	CA	ZF: (A)3 **(CA)18**	Tgu2: 42810182	ZF:	12	6	323	284	306 – 345
		CH: (A)3 **(CA)13**	Gga2: 67828480	CH:	4	2	323	294	323, 325
CAM-02	CA	ZF: **(CA)16**	Tgu7: 12381541	ZF:	11	9	373	341	365 – 389
		CH: **(CA)10** CG (CA)9	Gga7: 22132454	CH:	4	1	350	310	346
CAM-03	TG	ZF: [(TG)5TC]2 (TG)3TC **(TG)27**	Tgu7: 9747717	ZF:	12	11	209	123	168 – 269
		CH: (GA)2 CCTCCTC (TG)5 (TA)2 **(TG)14**	Gga7: 24391832	CH:	4	2	(164)	(111)	153, 163
CAM-04	GA	ZF: **(GA)11**	Tgu1: 34220431	ZF:	12	3	283	261	278 – 284
		CH: **(GA)11**	Gga1: 133721521	CH:	4	1	(275)	(253)	275
CAM-05	CA	ZF: **(CA)17**	Tgu1A: 45129155	ZF:	7	6	216	182	206 – 223
		CH: (CA)3 GACATA **(CA)12** (C)4 GGCCG (A)13 CAACC (A)14 C(G)4 (A)7	Gga1: 47660443	CH:	4	2	(198)	(109)	194, 197
CAM-06	AT	ZF: (AT)4 GT **(AT)8** TTATGT (AT)7	Tgu1A: 49994076	ZF:	8	5	284	190	283 – 295
		CH: **(AT)11** (W)4 G (TA)6 (W)13 G(T)3	Gga1: 52304006	CH:	4	1	278	190	278
CAM-07	CT	ZF: (CT)3 CC **(CT)17**	Tgu1A: 51267786	ZF:	12	6	234	153	233 – 265
		CH: (CT)6 CC **(CT)11**	Gga1: 53412026	CH:	3	1	234	166	235
CAM-08	TA	ZF: (T)6 **(TA)9** AA (TA)6	Tgu10: 3390752	ZF:	12	1	224	157	220
		CH: (T)5 **(TA)8** AA (TA)6	Gga10: 516461	CH:	4	1	(221)	(186)	219
CAM-09	GT	ZF: (GT)11	Tgu4A: 8999969	ZF:	11	8	325	303	314 – 324
		CH: (GT)14	Gga4: 17039238	CH:	4	(2) €	(324)	**(294)**	**(166, 193) **€
CAM-10	GT	ZF: (GT)22	Tgu13: 16024201	ZF:	11	8	201	157	183 – 210
		CH: (GT)15	Gga13: 1070809	CH:	2	1	(183)	(153)	186
CAM-11	GT	ZF: (GT)23	TguZ: 39096210	ZF:	12	6	147	101	145 – 157
		CH: (GT)11	GgaZ: 7888318	CH:	4	1	123	101	117
CAM-12	CA	ZF: (CA)20	Tgu2: 70094313	ZF:	12	9	370	330	371 – 433
		CH: (CA)2 GA (CA)2 CGCGTG (CA)2 CG (CA)3 TA **(CA)13**	Gga2: 62785492	CH:	3	2	(346)	(290)	346, 348
CAM-13	TC	ZF: (A)26 G(A)3 G(A)4 G(A)5 G(A)3 G(A)5 GCAAC (TG)2 (TC)6 TT **(TC)12** C(T)10	Tgu6: 26899281	ZF:	12	7	233	106	225 – 232
		CH: (TC)5 T **(TC)16** (C)4 (T)13	Gga6: 28449965	CH:	4	1	229	101	223
CAM-14	CA	ZF: **(CA)24** TG (CA)6	Tgu9: 5387194	ZF:	12	8	365	136	346 – 377
		CH: **(CA)13**	Gga9: 5323789	CH:	4	2	353	327	352, 354
CAM-15	GA	ZF: (GA)13	Tgu1A: 61859791	ZF:	12	3	266	240	260 – 266
		CH: (GA)7 GG (GA)2 GG **(GA)13**	Gga1: 73032096	CH:	4	2	(273)	(178)	247, 249
CAM-16	CA	ZF: (CA)16	Tgu17: 4369074	ZF:	11	5	290	258	287 – 301
		CH: (CA)15	Gga17: 4598995	CH:	3	1	(310)	(280)	301
CAM-17	TG	ZF: (T)9 G(GT)4 CC (TG )2 (TC)3 **(TG)12**	Tgu3: 2816652	ZF:	12	6	209	132	205 – 218
		CH: (T)3 **(TG)14** (CG)4 (TG)2 CGG (TG)4	Gga3: 10551236	CH:	3	2	207	153	204, 208
CAM-18	TA & TG	ZF: **(TA)11** T(TA)5 **(TG)7** & (AT)6	Tgu3: 31630754	ZF:	12	6	342	159	336 – 348
		CH: **(TA)10** T (TA)5 **(TG)11** & (TA)4	Gga3: 31888225	CH:	2	1	347	185	348
CAM-19	GT	ZF: (GA)3 (GT)6 TT **(GT)9**	Tgu1: 112898014	ZF:	12	6	231	180	227 – 248
		CH: (T)3 **(GT)20**	Gga1: 199733800	CH:	4	1	228	156	227
CAM-20	AT	ZF: (AT)5 TT **(AT)11** & (A)12 G(A)7	Tgu24: 5214087	ZF:	12	6	194	61	185 – 193
		CH: (AT)3 AA **(AT)9** & (AT)5 & (A)14	Gga24: 2939427	CH:	2	1	187	75	182
CAM-21	TG	ZF: (TG)13	Tgu2: 2028140	ZF:	12	4	277	251	265 – 274
		CH: (TG)12	Gga2: 2408229	CH:	4	1	(287)	(263)	287
CAM-22	GT	ZF: (A)8 &**(GT)13**	Tgu18: 10770012	ZF:	12	5	137	95	134 – 152
		CH: (A)5 & (A)6 &**(GT)12**	Gga18: 6214289	CH:	4	2	(134)	(88)	126, 131
CAM-23	TG	ZF: **(TG)18** (AG)5 GC (AG)3	Tgu6: 30010998	ZF:	12	5	147	93	140 – 151
		CH: (TG)5 TC **(TG)11** TT (AG)9	Gga6: 31243934	CH:	4	1	(147)	(93)	149
CAM-24	CA	ZF: (CA)3 (CG)2 **(CA)13**	Tgu1A: 1456627	ZF:	12	6	119	86	111 – 125
		CH: (GA)4 (CA)2 CG (CA)2 CG CACT **(CA)15**	Gga1: 2092872	CH:	4	1	121	67	111

*bp*, base pairs

*ZF*, zebra finch *Taeniopygia guttata*;

*CH*, chicken *Gallus gallus*;

*β*, The repeats shown in bold indicate those possessing the longest string of uninterrupted dinucleotide repeats;

*Sp*, species;

*Exp. length in ZF or CH (bp)*, expected PCR product size based on the pure zebra finch (ZF) or pure chicken sequence (CH);

^, those expected allele sizes in parentheses assume that a product is amplified in spite of the additional mismatches between the primer bases and the chicken genome.

*Minimum expected allele size in ZF or CH (bp)*, is based on the same sequences as above but after the deletion of the repeat region and repeat-like regions;

*n*, number of individuals genotyped (of species stated);

*#A*, number of alleles observed in the individuals genotyped;

€, same two alleles amplified in all individuals. Based on difference between the expected and observed allele sizes we suspected a different locus is amplifying in chicken;

### Cross-species amplification and polymorphism

The 24 primer sets developed were used to genotype a minimum of four individuals from each of eight species of Passeriformes and one species each of Ciconiiformes (Charadriiformes), Strigiformes, Coraciiformes and Galliformes (including zebra finch and chicken; classification following Sibley & Monroe [25]). The species tested covered a wide range of genetic distances from the zebra finch (species identities and sample sizes are provided in Table 4).

**Table 4:** Details of the 12 species tested and a summary of utility of the Conserved Avian Microsatellite (CAM) markers*

**Species**	**Status**	**Sample type and storage**	**Gen dist to ZF (ΔT**_**m**_**H)**	**Gen dist to CH (ΔT**_**m**_**H)**	**Order & Family ([**[25]**] / NCBI Taxonomy Database)**	**PCR profile**	**Pop**	**Loci amp. (%)**	**Loci poly. (%)**	**Geno-typer**	**Samples taken and DNA extracted by**	**Sample supplier(s)**
NEOGNATHAE												
**Passerines**												
Zebra finch	Captive	T/E &	0	28	Passeriformes	56	1	100	92	ADB	Jayne Pellatt,	Tim Birkhead
*Taeniopygia guttata*		B/E			Passeridae/Estrildidae						Jon Chittock	
Berthelot’s pipit	Wild	B/E	8.3	28	Passeriformes	56	4	96	70	LGS	LGS	David Richardson,
*Anthus berthelotii*					Passeridae							Juan Carlos Illera
House sparrow	Wild	B/E	8.5	28	Passeriformes	56	1	96	78	ADB	Nancy Ockendon	TB
*Passer domesticus*					Passeridae							
Chaffinch	Wild	B/E	10.0	28	Passeriformes	TD1	1	96	83	JP	Ben Sheldon	Ben Sheldon
*Fringilla coelebs*					Fringillidae							
Eurasian bullfinch	Wild	B/E	10.0	28	Passeriformes	TD1	1	96	65	JP	Kate Durrant,	Tim Birkhead
*Pyrrhula pyrrhula*					Fringillidae						Stuart Sharp, Simone Immler	
Great tit	Wild	B/E	11.1	28	Passeriformes	TD1	1	96	56	JP	Louise Gentle,	TB
*Parus major*					Paridae						Harrie Bickle	
European blackbird	Wild	B/E	11.7	28	Passeriformes	TD1	1	83	60	JP	Michelle Simeoni	Ben Hatchwell
*Turdus merula*					Muscicapidae/Turdidae							
Rifleman	Wild	B/E	19.7	28	Passeriformes	56	1	96	61	SAJP	SAJP	Ben Hatchwell
*Acanthisitta chloris*					Acanthisittidae							

**Non-passerines**												
Leach’s storm-petrel	Wild	B/E	21.6	28	Ciconiiformes	56	4	96	56	AWJB	AWJB	AWJB
*Oceanodroma leucorhoa*					Procellariidae							
Barn owl	Wild	B/E	22.5	28	Strigiformes	TD1	1	92	32	JP	Akos Klein	Akos Klein
*Tyto alba*					Tytonidae							
European roller	Wild	B/E	25.0	28	Coraciiformes	B,	1	96	39	DM-G,	DM-G	Deseada Parejo,
*Coracias garrulus*					Coraciidae	TD2				MM-M		Jesus Avilés
PALAEOGNATHAE												
Chicken (domestic)	Captive	B/E	28.0	0	Galliformes	TD1	1	100	38	JP	Hans Cheng	Hans Cheng
*Gallus gallus domesticus*					Phasianidae							

*Four individuals were tested per species with 24 Conserved Avian Microsatellite (*CAM*) primer sets. All PCR failures were rechecked for amplification by a different researcher (GJH) using the touchdown PCR program (TD1);

PCR profiles:

A: QIAGEN Multiplex PCR Master Mix; 95°C for 15 minutes, followed by 35 cycles of 94°C for 30 seconds, 56°C for 90 seconds, 72°C for 1 minute, and finally 60°C for 30 minutes.

B: (used only for the unrelated rollers), Bioline DNA *Taq* polymerase, 94°C 3 min, then 35 cycles of 94°C for 30 s, 56°C for 30 s, 72°C for 30 s, and finally 72°C for 10 min.

TD1: QIAGEN Multiplex PCR Master Mix; touchdown PCR program, 95°C for 15 min followed by 16 cycles of 94°C for 30 s, 65°C for 90 s decreasing by 1°C per cycle, 72°C for 60 s for 10 cycles, followed by 94°C for 30 s, 55°C for 90 s, 72°C for 60 for 25 cycles, with a final step of 72°C for 10 min.

TD2: (used only for the related European rollers) Bioline DNA *Taq* polymerase, touchdown PCR profile, 94°C for 3 min, then 10 cycles of 94°C for 30 s, 65°C for 30 s (and decreasing by 1°C for 15 cycles), 72°C for 1 min, followed by 28 cycles of 94°C for 30 s, 50°C for 30 s and 72°C for 30 s, followed by one cycle of 5 min at 72°C.

T, tissue; B, blood; E, ethanol; Pop., number of populations represented in the four individuals tested; amp., amplifying; poly., polymorphic; Loci poly. (%) indicates the proportion of loci polymorphic of those amplifying.

Genetic distance to ZF, genetic distance from species tested to zebra finch based on [33] and the classification of [25]; Genetic distance to CH, genetic distance from species tested to chicken [33].

All individuals had been sampled in the wild with the exception of the zebra finch and chicken individuals (Table 4). The latter were sampled from captive populations maintained at the University of Sheffield and the United States Department of Agriculture (Agriculture Research Service, East Lansing, USA), respectively. For each species, all individuals genotyped were unrelated as known, except for the chicken and European rollers. All four chicken were siblings and three of the European rollers were siblings. The chicken individuals genotyped were four siblings from the East Lansing mapping population, which consists of fifty-two BC1 animals derived from a backcross between a partially inbred jungle fowl line and a highly inbred white leghorn line [49]. These individuals, therefore, will display a maximum of four alleles per locus, but often fewer. Additionally, a higher proportion of the chicken siblings might be expected to be heterozygous than in a wild population because the mother and father of the chicken pedigree originated from different breeds. Polymorphism in chickens at the *TG* and *CAM* loci was omitted from analyses for three reasons: (1) the chicken individuals tested belonged to a backcrossed mapping pedigree; (2) all the other species tested were comparable, being all at a genetic distance of 28 from chicken (genetic distance: DNA: DNA melting temperature (Δ T_m_) hybridisation distance, [33]) and, finally, (3) the primer sets had been engineered more specifically to amplify in chicken than in the other species tested. The European rollers genotyped initially included four nestlings sampled from two nests (including three siblings from one nest). When the loci that failed to amplify were rechecked, unrelated European roller individuals were used. All individuals genotyped were sampled from a single population, except the Leach’s storm-petrels, for which the six individuals were sampled from four populations, and Berthelot’s pipits, for which each of the four individuals sampled was from a different population.

Approximately 20–50 μl of blood was collected from each individual and stored in 1.5 ml of absolute ethanol in rubber-sealed screw-topped microfuge tubes. Genomic DNA was extracted using an ammonium acetate precipitation method [50] or a salt extraction method [51]. Each DNA extraction was tested for amplification and sex-typed using the *Z-002*[52] or (for the Berthelot’s pipit and the European roller) *P2*/*P8*[53] sex-typing markers.

Each primer set was tested in isolation (single-plexed) in all species. Primer sets (using the zebra finch version of the primer sequence) were checked for their potential to form hairpins and to identify any PCR incompatibilities due to primer sequence similarity using AUTODIMER software [54], http://www.cstl.nist.gov/strbase/software.htm) using a ‘conservative minimum threshold score’ of seven.

Single-plex PCR reactions were performed in 2-μl volumes using QIAGEN Multiplex PCR Master Mix (QIAGEN Inc.) for all species except the European roller and its reruns. Each 2-μl PCR contained approximately 10 ng of lyophilised genomic DNA, 0.2 μM of each primer and 1 μl QIAGEN Multiplex PCR Master Mix [55]. For all species, PCR amplification was performed in the same laboratory in Sheffield using a DNA Engine Tetrad 2 thermal cycler (model PTC, MJ Research, Bio-Rad, Hemel Hempstead, Herts, UK). PCR amplification was performed using an annealing temperature of 56°C or a touchdown PCR program (Table 4). Slightly different PCR protocols were used for some species, since they were performed by different researchers at different times and using different DNA *Taq* polymerases (Table 4). However, these differences are not expected to have any measurable effect. The European roller amplifications were performed in a 10-μl PCR reaction that contained approximately 20 ng of genomic DNA, 0.5 μM of each primer, 0.2 mM of each dNTP, 2.0 mM MgCl_2_ and 0.25 units of *Taq* DNA polymerase (Bioline) in the manufacturer’s buffer (final concentrations: 16 mM (NH_4_)_2_SO_4_, 67 mM Tris–HCl (pH 8.8 at 25°C), 0.01% Tween-20). Products were diluted 1 in 500 prior to separation on an ABI 3730 48-well capillary DNA Analyser and allele sizes were assigned using GENEMAPPER v3.7 software (Applied Biosystems, California, USA). The same DNA Analyser at Sheffield was used for separating the amplified products for all species. Alleles were scored separately for each species, using species-specific allele bin sets, in different sessions by different researchers but in the same laboratory and using the same methods (details in Table 4).

Previous work has identified that it is worth retesting any markers that fail to amplify at the first PCR attempt [21]. All markers that failed to amplify were therefore rechecked by performing a repeat PCR and the majority amplified at the second PCR attempt. When the 24 markers were initially tested, a maximum of six markers (25%) failed to amplify in a single species; however, the majority amplified at the second PCR attempt (Table 4 and Additional file 1).

For four species, Berthelot’s pipit, rifleman, Leach’s storm petrel and European roller, a proportion of the *CAM* and *TG* loci [21] were assessed in a larger sample of unrelated individuals (*n* = 17–30) from a single population in order to check for Hardy–Weinberg equilibrium and estimate null allele frequencies (calculated using GENEPOPv4.0.10, [56] and CERVUSv3.0.3, [57]). The characteristics of the *CAM* and *TG* marker sets were then compared for these four species, in terms of the number of loci deviating from Hardy–Weinberg equilibrium and the proportion possessing high null allele frequency estimates.

All statistical analyses were carried out in R version 2.14.1 [58]. Differences in the proportions of polymorphic loci across passerines and non-passerines, and between *CAM* and *TG* loci, were tested using chi-squared (χ^2^) tests. Linear regression was used to test for whether the percentage of polymorphic loci per species was related to the genetic distance from zebra finch.

## Results and discussion

### Identification of microsatellite sequences in the zebra finch and chicken genomes

There were similar total numbers of dinucleotide microsatellite sequences of eight or more repeats in the zebra finch and chicken genomes (6,458 versus 6,581, respectively; Table 1). Hits to the “unknown” chromosome were not included, since duplicate sequences have been observed on both the named chromosomes and the ‘unknown’ chromosome and these occurrences are probably artefacts of the assembly process (DAD pers. obs.). It should also be noted that a male was sequenced to obtain the zebra finch genome, whereas a female was used for the chicken, so that only the chicken genome includes sequence derived from the W chromosome. However, due to the small size of the W chromosome (representing only 0.02% of the assembled chicken genome), its inclusion is not expected to influence significantly the total number of microsatellites detected.

Only one chicken and no zebra finch microsatellites were found that contained a GC/CG motif, suggesting that these motif types are rare and/or shorter than eight units in length in the avian genome. Although the total numbers of microsatellite loci were similar between the zebra finch and chicken, the zebra finch possessed a higher proportion of AT/TA repeats, and fewer CA/GT and GA/CT motifs, than chicken (Table 1; heterogeneity test, χ^2^ = 381.6, d.f. = 2, *p* < 0.0001). These differences were unexpected and the reasons for them are currently unknown.

### Identification of highly conserved microsatellite loci

Forty-two homologous microsatellite loci were identified in both the zebra finch and chicken, with each pair having a BLAST E-value better than E-59. None of these newly identified conserved sequences matched any of the conserved EST-based microsatellite loci for which primer sets had already been developed by Dawson *et al*. [21]. The conserved loci possessed the following dinucleotide motifs: CA/GT motif (n = 22), AT/TA (n = 16) and GA/CT (n = 4). The distribution of motif types in the conserved loci did not differ from expectation based on their frequencies in the zebra finch (heterogeneity test, χ^2^ = 5.42, d.f. = 2, *p* = 0.07) or chicken genome (heterogeneity test, χ^2^ = 2.95, d.f. = 2, *p* = 0.23; Table 1). All 42 zebra finch sequences were aligned with their chicken homologues in an attempt to create a consensus hybrid sequence.

### Creation of a consensus hybrid sequence and primer design

Consensus primer sets were created for 24 of the 42 unique loci identified (57%) using the primer design criteria outlined above (Tables 1 &2; full sequences of the loci are provided in Additional file 2). In contrast to Dawson *et al*. [21], we were not able to create primer sets that were always 100% homologous to chicken but all matched 100% to zebra finch, and were at least 86% similar to their homologous chicken sequences (by including 1–2 degenerate bases in 25 primers). Only a single degenerate base in just one primer was required in the earlier EST study, which then matched 100% to both species (34 primer sets; [21]). Many more degenerate bases were used in the *CAM* marker set than in the earlier *TG* marker set (*CAM*: 28 degenerate bases spread over 18 of the 24 markers; *TG*: one degenerate base in one of the 34 markers; this study versus Dawson *et al*. [21]). Only six *CAM* consensus sequences contained regions of microsatellite-flanking sequence that were identical in zebra finch and chicken for a sufficient length from which to design primers without using any degenerate bases (*CAM-06*, *CAM-13*, *CAM-17*, *CAM-18*, *CAM-20* and *CAM-24*; Table 2). The remaining 18 primer sets contained between 1–2 degenerate bases per primer sequence (a maximum of 3 degenerate bases per primer pair) and, of these, only six were 100% matches to both zebra finch and chicken, when accounting for the degenerate bases used. We attempted to design the most consensus primers we could. The primer sequences of the remaining 12 degenerate primer sets were a 100% match to zebra finch and a match to chicken of between 86–96%.

As expected, all 24 loci possessed dinucleotide motifs in chicken and zebra finch, with the majority being the CA/GT motif (n = 16), although some had AT/TA (n = 4) and GA/CT (n = 4) motifs. The same motif type was present in both chicken and its zebra finch homologue at all 24 loci (Table 3). Most loci possessed several different dinucleotide repeat regions and some also possessed additional mononucleotide repeat regions in the sequence (Table 3). When the longest string of uninterrupted dinucleotide repeats at each orthologous locus was compared between chicken and zebra finch there was a significant difference in the number of repeat units (paired t-test, t = 2.18, d.f. = 23, *P* = 0.04; 15 loci had fewer repeats in chicken, six had more and three the same number of repeat units; Table 3). The 24 selected loci possessed a minimum of eight uninterrupted dinucleotide repeat units (in both species) and a maximum of 27 in zebra finch and 20 in chicken (Table 3).

No hairpins were detected in any primer sequences when analysed using only the pure zebra finch version of each primer (assessed using AUTODIMER software). Three pairs of primer sequences displayed some degree of similarity and should be avoided as potential multiplex combinations to prevent the risk of forming primer dimers (CAM-02R–CAM-15R, CAM-03R–CAM-20F and CAM-05R–CAM-06R). However, the check for primer similarity (using AUTODIMER software) is of limited utility when checking primers containing degenerate bases because the degenerate bases are regarded as unknown bases and some unidentified primer pairs may turn out to be incompatible. We therefore recommend typing the loci both singly and in multiplex PCR reactions to confirm that the genotypes match before routinely using any multiplex set, especially when the primer sequences contain degenerate bases. When up to three degenerate bases are used, as in this study, the maximum number of forward and reverse sequence combinations per primer set is eight and the resulting variation in annealing temperatures between the forward and reverse primers might potentially cause PCR amplification problems. We recommend designing primer sets for standard microsatellite loci using PRIMER3 with a maximum difference between the forward and reverse primer melting temperature of 0.5°C. However, a difference of up to 2°C has been found to be acceptable for the amplification of many primer sets (e.g. [59]). Unreliable PCR amplification of these loci is most likely in the non-passerine species, as they are more genetically distant from zebra finch and are therefore more likely to exhibit base mismatches in the primer binding regions. Incomplete PCR amplification can be identified by testing a range of annealing temperatures, performing repeat PCRs and/or the typing of a pedigree (if available), and, if detected, can be improved by PCR optimisation methods.

### Homology to expressed and coding sequence

Highly conserved microsatellites have been successfully isolated from ESTs [21]. The majority of the 24 *CAM* sequences (17/24) were found to be homologous to avian ESTs, avian (or mammalian) mRNA sequences or known genes (identified by sequence similarity searches of the GenBank nr, EST (“EST_others”) nucleotide databases and the zebra finch and chicken genomes; Table 2). Some of the microsatellite sequences were located within exons, which may explain why these sequences are conserved among many species.

### Genome locations and linkage

All 24 loci could be assigned a location in both the zebra finch and chicken genome based on sequence similarity. Twenty-three loci were assigned to an autosomal location and one locus (*CAM-11*) was assigned to the Z chromosome in both species (Figure 1). Two pairs and one triplet of loci were assigned locations less than 5 Mb apart in both the chicken and zebra finch genomes; there is therefore an increased possibility of these loci being in linkage disequilibrium because recombination rates between them will be relatively low: *CAM-02–CAM-03* on Gga7/Tgu7*, CAM-05–CAM-06–CAM-07* on Gga1/Tgu1A and *CAM-13–CAM-23* on Gga6/Tgu6 (Figure 1). Several *CAM* loci were typed in a pedigree of over 300 house sparrows (JS *et al*. unpublished data). This analysis confirmed, as expected, that loci *CAM-05*, *CAM-06* and *CAM-07* were all linked. Additionally, loci *CAM-01* and *CAM-12* were also linked in the house sparrow linkage map (JS *et al*. unpublished data; both loci located on chromosome 2 in zebra finch (27 Mb apart) and chicken (5 Mb apart), Figure 1). Loci *CAM-02* and *CAM-13* were not typed in the house sparrow pedigree so could not be checked for linkage to the other locus located on the same chromosome (*CAM-03* and *CAM-23* respectively).

### Cross-species amplification

**Figure 2:**
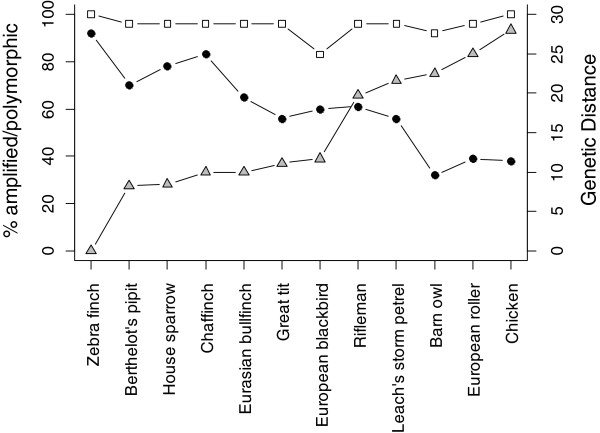
**Percentage of *CAM *loci amplified (white squares) and polymorphic (black circles), alongside genetic distance from the zebra finch (grey triangles) for 12 species.** % Polymorphic, proportion of loci polymorphic of those amplifying for each set of loci. Four individuals were genotyped for each species at 24 loci. Genetic distance, DNA:DNA Δ T_m_ hybridisation distance [33].

All loci amplified in both zebra finch and chicken (Tables 3 &4, Figure 2). The ranges of allele sizes obtained by genotyping zebra finches and chickens were close to those expected based on the respective genome sequences, with the exception of locus *CAM-09* in chicken. The maximum difference between the expected allele size and the allele size range observed for each species was 11 bp (except *CAM-09* in chicken; Table 3); since the source genome sequence was isolated from an individual belonging to a different population to the individuals genotyped, small allele size differences (such as 1–20 bp) are expected. Locus *CAM-09* was 101 bp smaller in size in chicken than expected, however, this marker remains of potential utility in other species. We suspect that a deletion may have occurred in the chicken (breed/population) genotyped, or that a different locus is being amplified, possibly due to poor similarity of the *CAM-09* primer sequences to chicken (three degenerate bases were used (one in the forward primer and two in the reverse) but, despite this, three bases in the forward primer and two in the reverse still did not match chicken 100%; Table 2). It was surprising that, despite up to three chicken–primer base mismatches per primer sequence (in addition to the presence of up to two degenerate bases), and the differences in primer annealing temperatures in different species caused by this (Additional file 3), all the primer sets amplified in chicken. Amplification may have been assisted by the use of a touchdown PCR program and the use of the QIAGEN Multiplex PCR Master Mix, which enhances the likelihood of successful PCR amplification from primers with differing annealing temperatures. For the majority of loci (including *CAM-09*), the sizes of the alleles observed in the ten other species tested were very similar to those expected and observed in zebra finches (and/or chickens, except *CAM-09*) (Additional file 1). It is expected that for each species a few loci will not possess high sequence similarity and, because the identity of those not possessing sequence similarity is different in each species, this does not present a problem. We compared sequences to the recently released collared flycatcher (*Ficedula albicollis)* and budgerigar (*Melopsittacus undulates*) genome sequences (http://www.ensembl.org/index.html; Dawson *et al*. unpublished data). A homologue was identified in each case and all contained a microsatellite repeat (including *CAM-09*; *CAM-24* cannot be checked because it cannot be identified in the available assemblies). This suggests the correct target locus was being amplified in the majority of species–marker tests.

The degree of sequence similarity between distantly related species affects the range of species that will amplify [60]. Those markers designed from sequences with high similarity between distantly related species (i.e. those with an E-value of E-80 or better between zebra finch and chicken) have been found to amplify in virtually all birds [21]. Dawson *et al*. [21] used a different BLAST program (WU-BLAST) when assessing loci for potential cross-species utility. However, the BLAST E-values obtained via WU-BLAST and NCBI BLAST (as used for this study) for the same sequence are normally very similar (DAD unpublished data). During this study we utilised sequences with a lower similarity between zebra finch and chicken (those displaying a BLAST E-value better than E-59). This weaker cut-off was necessary to enable the identification of homologous sequences that possessed eight repeats in *both* zebra finch and chicken but the trade-off was that in most cases the poorer similarity made it impossible to design primers that were a complete match to both zebra finch and chicken. The reduced primer similarity to chicken was expected to lower the utility of these markers in species distant to zebra finch but it was hoped that, for those species close to zebra finch (passerines), a high number of polymorphic loci would be identified. On average, 94% of loci amplified in each of the seven passerine species tested (range 83–96%) and 95% amplified in each of three non-passerine species (range 92–96%; zebra finch and chicken data excluded, Table 4, Figure 2). The number of loci that amplified within each species was not related to their genetic distance from the zebra finch (Figure 2).

### Cross-species polymorphism

Of the *CAM* loci that amplified, 56–83% (mean 68%) were polymorphic in each passerine compared to 32–56% (mean 42%) in each non-passerine, and this difference was significant (zebra finch and chicken data excluded; χ^2^ = 6.42, d.f. = 1, *P* = 0.01; Table 4). Additionally, more of the amplifying *CAM* loci were polymorphic than the amplifying *TG* loci ([21]; zebra finch and chicken data excluded; χ^2^ = 7.81, d.f. = 1, *P* = 0.005). Of the *TG* loci that amplified, 24–76% (mean 47%) were polymorphic in a passerine species and 18–26% (mean 22%) in a non-passerine species [21]. When assessed in a minimum of four individuals per species, the species with the highest proportion of polymorphic *CAM* loci was, as expected, the zebra finch (92%), followed by the chaffinch (*Fringilla coelebs*; 83%), while the lowest proportion in a passerine was 56% in the great tit (*Parus major*; Table 4, Figure 2).

**Figure 3:**
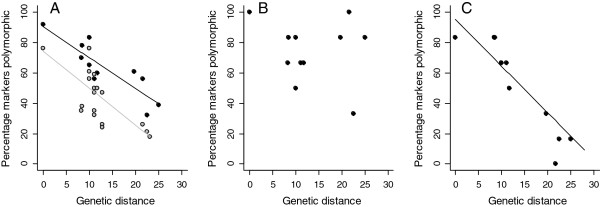
**Percentage of *CAM *(black) and *TG *(grey) microsatellite markers polymorphic in relation to genetic distance from zebra finch. A:** All 24 *CAM* markers included (*CAM* = this study; *TG* = Dawson *et al*. [21]); **B:** Six *CAM* markers with 100% primer sequence similarity to chicken (and zebra finch); **C:** Six *CAM* markers with poor primer sequence similarity to chicken (but 100% identical to zebra finch). Percentage markers polymorphic, proportion of loci polymorphic of those amplifying for each set of loci (*CAM* and *TG* sets). Genetic distance, DNA:DNA Δ T_m_ hybridisation distance [33]. Four individuals were genotyped at 24 loci for each of the 11 species (including zebra finch *Taeniopygia guttata* but excluding chicken *Gallus gallus*; see text).

**Figure 4:**
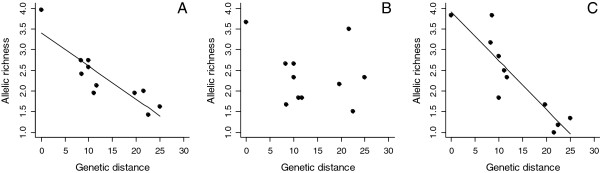
Allelic richness (mean number of alleles per polymorphic locus) of the *CAM *markers in relation to genetic distance from zebra finch.* **A:** All 24 *CAM* markers included; **B:** Six *CAM* markers with 100% primer sequence similarity to chicken (and zebra finch); **C:** Six *CAM* markers with poor primer sequence similarity to chicken (but 100% identical to zebra finch). Genetic distance, genetic distance of the genotyped species from zebra finch (*Taeniopygia guttata*) DNA:DNA ΔT_m_ hybridisation distance [33]. *Four individuals were genotyped at 24 loci for each of 11 species (including zebra finch *Taeniopygia guttata* but excluding chicken *Gallus gallus*; see text).

**Figure 5:**
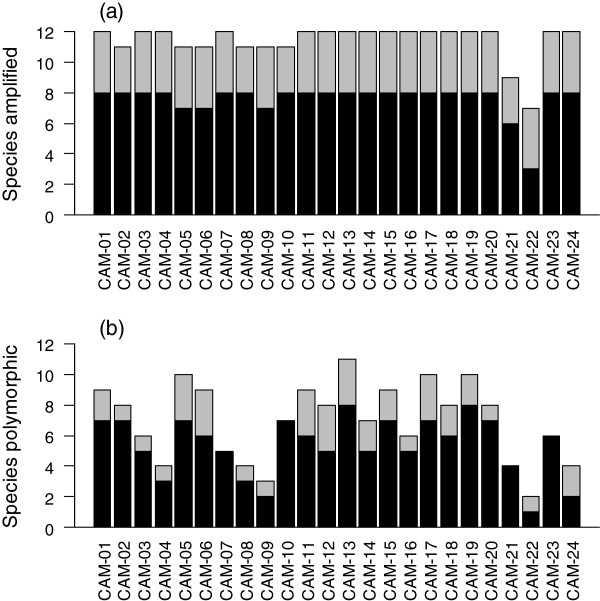
**Number of species (A) amplified and (B) polymorphic at each individual *CAM *locus.** Black bars represent passerines and grey bars non-passerines. Each locus was tested in 12 species (including zebra finch *Taeniopygia guttata* and chicken *Gallus gallus*), which included 8 passerine species, and 4 non-passerine species. Classification of species as passerine or non-passerine was following Sibley & Monroe [25]. The data presented is based on the genotyping of 4 individuals per species. For details of which species failed to amplify see Additional file 1.

When all 24 *CAM* markers were considered as a whole, the proportion of loci polymorphic per species was negatively correlated with genetic distance from the zebra finch (Figure 2), as was also previously found for the *TG* loci [21], despite the fact that the *CAM* loci displayed a repeat region of at least eight repeat units in chicken (chicken excluded; *CAM* loci: *F* = 27.55, d.f. = 1, 9, *R*^2^ = 0.73, *P* = 0.0005; *TG* loci: *F* = 15.30, d.f. = 1, 17, *R*^2^ = 0.44, *P* = 0.001; Figure 3A). Additionally, the mean number of alleles per polymorphic locus decreased with increasing genetic distance from the zebra finch (chicken excluded; *F* = 22.99, d.f. = 1, 9, *R*^2^ = 0.68, *P* < 0.001; Figure 4A). These regressions remained significant after controlling for differences between passerines and non-passerines, and when a phylogenetic correction was used (data not shown), indicating that the effect of genetic distance on polymorphism was a linear, rather than group effect. Approximately 20% more of the loci that amplified were polymorphic per species than was achieved previously by studies attempting to create conserved avian microsatellite loci. Each marker displayed a varying degree of cross-species utility (Figure 5, Additional file 4), possibly due to the differing degree of primer sequence similarity to chicken (Table 4, Additional file 3). In order to investigate this, we selected two subsets of six *CAM* markers: (Set 1) those that were a 100% match to chicken (and zebra finch) and possessed no degenerate bases (*CAM-06*, *CAM-13*, *CAM-17*, *CAM-18*, *CAM-20* and *CAM-24*) and (Set 2) those which displayed poor similarity to chicken (but a 100% match to zebra finch; *CAM-03*, *CAM-04*, *CAM-10*, *CAM-15*, *CAM-21* and *CAM-23*) and analysed these two groups separately. For Set 1 (the highly conserved markers), there was no relationship between the percentage of species polymorphic and genetic distance from zebra finch (linear regression: *R*^*2*^ = 0.11, d.f. = 10, *P* = 0.15, zebra finch and chicken excluded; Figure 3B). This appears to be a result of more markers in this set being polymorphic in those species distant to zebra finch (Figure 3B). However, in Set 2 (the more weakly conserved markers), the percentage polymorphism declined significantly with genetic distance from zebra finch (linear regression: *R*^*2*^ = 0.75, d.f. = 10, *P* = 0.0002, zebra finch and chicken excluded; Figure 3C). Set 2 also displayed a decrease in the mean number of alleles with increasing genetic distance from zebra finch (*R*^*2*^ = 0.8, d.f. = 10, *P* = 0.0002; Figure 4C), whereas in Set 1 there was no such fall (*R*^*2*^ = 0.07, d.f. = 10, *P* = 0.42; Figure 4B). In order to identify why markers with poor primer sequence similarity to chicken displayed a fall in variability as genetic distance increased, we checked both sets of loci for sequence similarity with the collared flycatcher and budgerigar genome sequences. These species are both useful for this investigation because their genetic distance from chicken is the same as the other species used in this study (genetic distances (Δ T_m_): collared flycatcher–chicken = 28 and budgerigar–chicken = 28; collared flycatcher–zebra finch = 11.7 and budgerigar–zebra finch = 23.1; [33]). We checked how many bases in each primer sequence mismatched with their zebra finch and chicken homologue and how the repeat regions varied between the species. This revealed that for both Set 1 and Set 2, only two and one primer sets completely matched flycatcher respectively, but the number of bases mismatching in each primer set was quite low in both groups (a maximum of three mismatches per primer set, except for *CAM-06* and *CAM-21*). In the more distant budgerigar, when the weakly-conserved markers of Set 2 were analysed, there were more mismatches per primer set than observed in the flycatcher: four markers had over three bases mismatching per primer set, one marker had one mismatch and for only one marker did both the forward and reverse primer sequences completely match budgerigar. Whereas, in the strongly-conserved marker Set 1, for the five homologous loci that could be identified (i.e. except *CAM-24*) all primer sets were a complete match to budgerigar. It was surprising that the primer sequences of the markers in Set 1 displayed higher similarity to budgerigar than flycatcher. All loci in both sets contained at least five uninterrupted repeats both species, except *CAM-03* in budgerigar (*CAM-24* could not be checked). There was no relationship between the mean number of repeats possessed and the number of bases mismatching in the primer sequences (mean number of repeats in Set 1 versus Set 2, flycatcher: 11 versus 11, budgerigar: 6 versus 7). This suggests that primer sequence similarity is the main factor affecting the identification of a polymorphic locus in this set of 24 *CAM* markers. Based on the number of repeats observed in budgerigar, other *CAM* loci would be expected to be polymorphic in non-passerines but the primers appear to be amplifying only one of the alleles (19 loci had more than 5 repeats in budgerigar and a maximum of 11 repeats observed; *CAM-24* could not be checked). Perhaps, in distantly related species, mismatches between the target sequence and primer sequence result in amplification failure of some alleles due to large differences in the melting temperatures between the forward and reverse primer and between these and the PCR annealing temperature used. These base mismatches and mismatched melting and annealing temperatures may lead to only a single allele (with highest similarity to the primers) being amplified during the PCR. It is unclear why the primer set does not simply fail to amplify a product but perhaps the use of QIAGEN Multiplex PCR Master Mix reaction buffer enables amplification even when a primer set has poor similarity to the target. Alternatively, perhaps those displaying poor similarity to chicken are amplifying a different (invariant) locus in many of those species distant to zebra finch although this seems unlikely based on the agreement between the observed and expected allele size for each locus. The six well-conserved markers in Set 1 for which the proportion of polymorphic loci did not decrease with genetic distance (i.e. *CAM-06*, *CAM-13*, *CAM-17*, *CAM-18*, *CAM-20* and *CAM-24*) are expected to be of highest utility in species most distant to zebra finch.

We deduce that there are several important factors for ensuring polymorphism across the widest range of species (and for avoiding null alleles) when designing conserved markers: (1) the most distantly related species possible should be selected for designing the primers; (2) the similarity of the homologous regions should be high (displaying a BLAST E-value of E-80 or better); (3) a minimum of 8 uninterrupted repeats should be present in each species’ sequence used in the alignment; (4) the primer sequence must match both/all species 100%; (5) the use of degenerate bases should ideally be avoided or else minimized (to no more than one degenerate base per primer set); (6) the forward and reverse primer melting temperatures should ideally be within 0.5°C of each other (maximum 2°C); and (7) when degenerate bases are used it is important to confirm that all the alternative states of the forward and reverse primers are compatible and ensure that the melting temperature of all alternative states are within 0.5°C of each other.

The *CAM* loci were of utility in non-passerine birds. The nearest avian order, in terms of genetic distance, to Passeriformes is the order Ciconiiformes (also known as Charadriiformes, shorebirds and allies, [33]). We tested one ciconiiform, the Leach’s storm-petrel, in which 23 (96%) loci amplified and 13 (56%) of those amplifying were found to be polymorphic (Table 4, Additional file 1, Figure 2). In the two species very distant from both zebra finch and chicken, the barn owl and European roller, most of the markers amplified (92–96%) and 32–39% of those amplifying were polymorphic; Table 4, Additional file 1, Figure 2). When tested in chicken, 38% of the loci (*n* = 9) were polymorphic (Tables 4, Additional file 1).

### Typical proportions of loci polymorphic among those amplifying in other studies

The levels of variability in each species when typed with the *CAM* loci might be affected by factors other than genetic distance, for example, genetic bottlenecks, founder effects, or long-term inbreeding, though we are unaware that these factors have affected any of the species/populations we typed. Additional polymorphic loci have been genotyped in the same three non-passerine species that we tested and this work did not suggest that any of the three species had exceptionally low variability (barn owl, [61]; Leach’s storm-petrel, [62]; European roller, [63]).

We found the proportions of *CAM* loci polymorphic among those amplifying to vary between 38–92% per species when all 24 loci were considered (Table 4; i.e. including those markers with good zebra finch–chicken primer sequence similarity and those loci in which it was poor). These figures are typical of those found in other studies. The proportion of loci polymorphic of those amplifying appears to vary widely among species (Additional file 5).

It is currently unclear if non-passerines are generally less variable than passerines. Further species need to be tested and more work performed to resolve this. If, however, the majority of non-passerine species do display lower variation than passerines then possible causes could be: (1) smaller effective population sizes in non-passerines, (2) higher microsatellite mutation rates in passerines compared to non-passerines or (3) different life histories between passerine and non-passerines. (1) Using a database for North American birds (Partners in Flight Landbird Population Estimates Database, http://rmbo.org/pif_db/laped/default.aspx, [64]), we found that passerines generally exhibited much larger population sizes than non-passerines (mean ± s.e. individuals per population = 15,524,224 ± 1,950,522 for passerines and 2,789,765 ± 835,772 for non-passerines; independent samples t-test, *t* = 5.83, d.f. = 383, *P* < 0.0001). The higher mean population size of passerines may lead to them retaining more genetic variability than non-passerines. (2) Microsatellite mutation rates vary among species [34]. Microsatellites may mutate more rapidly in passerines than non-passerines and, as a result, passerines are more variable. (3) The typically longer generation time of non-passerines [65] is expected to result in a lower evolutionary rate [66]. In contrast, non-passerines generally display lower levels of extra-pair paternity (EPP) than passerines [67]. A high rate of EPP will increase the variance in male reproductive success and reduce the effective population size (*N*_e_), and hence the level of genetic variability. However, the difference in male variance and the consequent effect on *N*_e_ will be relatively small.

### Individual marker performance

Nineteen loci were polymorphic in a minimum of 50% of the eight passerine species tested (when all loci were assessed in a minimum of 4 individuals/species; Figure 5, Additional file 1). The best performing loci in passerines were *CAM-13* and *CAM-19*, which were polymorphic in all eight passerine species tested (including zebra finch, Figure 5, Additional file 1). Seven further loci were polymorphic in seven of the eight passerine species tested (*CAM-01*, *CAM-02*, *CAM-05*, *CAM-10*, *CAM-15*, *CAM-17* and *CAM-20*, Figure 5, Additional file 1). The poorest performing locus, *CAM-22*, failed to amplify in five passerine species (however, all non-passerines amplified; Figure 5).

### Locus homology to bird EST/genic sequences

Seventeen of the 24 markers developed were homologous to a bird EST sequence and/or gene (all markers except *CAM-02*, *CAM-03*, *CAM-04*, *CAM-09*, *CAM-12*, *CAM-22* and *CAM-24*; Table 2, Additional file 6). Homology to bird EST/genic sequences, which are expected to be most conserved, did not reduce the number of species found to be polymorphic. In fact, the opposite was true: markers homologous to EST/genic bird sequences were more polymorphic across bird species (χ^2^ = 11.77, d.f. = 1, *P* = 0.006). This is in accordance with evidence from previous studies, which have failed to show that microsatellite markers developed from non-EST sequences are more variable than those from ESTs [37,38].

### Null alleles

For four species: Berthelot’s pipit, rifleman, Leach’s storm petrel and European roller, some of the polymorphic *CAM* and *TG* loci (n = 5–12) were additionally typed in 17–30 individuals from a single population and assessed for deviation from Hardy–Weinberg equilibrium and null allele frequencies estimated (Additional file 7). When the data from these four species was combined, there was no overall difference in the proportion of loci displaying high estimated null allele frequencies between the *CAM* and *TG* loci (χ^2^ =0. 0.001, d.f. = 1, *P* = 0.98; Additional file 7).

It is likely that null alleles will be more common in more distant species, especially when using primer sets that are less conserved (between chicken and zebra finch). If this happens, the amplified product could be sequenced and species-specific primer sets designed.

### Chromosome locations and sex linkage

All individuals genotyped with the *CAM* loci were of known sex based on plumage characteristics or PCR sex-typing. The individuals genotyped included both males and females for each species. Males (ZZ) of all species amplified at all loci, indicating that no *CAM* loci were purely W-linked in any species.

All the predicted genome locations of these loci were autosomal except for locus *CAM-11*, which was predicted to be Z-linked (Figure 1). Genotypic evidence supported the suggested Z-linked status of this locus in every species in which it was polymorphic: zebra finch, house sparrow, Berthelot’s pipit, chaffinch, Eurasian bullfinch, rifleman, European roller and Leach’s storm-petrel (Additional file 1). All females were hemizygous whereas at least some males were heterozygous, 5–28 males and 3–22 females per species (regarding Leach’s storm-petrel, see below). In Leach’s storm-petrels, *CAM-11* amplified both W *and* Z-linked alleles and could be used to sex-type individuals. Females were hemizygous, displaying one allele of size 113 bp (n = 22 females) and males were heterozygous or homozygous with observed allele sizes of 134, 136, 138 and 145 bp (n = 26 males). This suggests that the 113-bp allele is located on the W chromosome and the 134–145-bp alleles are located on the Z chromosome. The absence of an amplified Z-allele in females suggests that the 113-bp W allele is amplified in preference to the Z alleles that must also be present. This is expected to happen, for example, if the primers are a better match to the W locus than the Z locus. Upon re-examination, very weak Z alleles (peak heights of 97–288 relative fluorescence units (RFU)) were seen in some female chromatographs, supporting this hypothesis. These weakly-amplified female Z alleles were only observed when the peak height of the W allele was well over 2000 RFU (most over 6000 RFU) and they often failed to amplify at all when the sample was rerun. Locus *CAM-11* may prove suitable for sex-typing other related species of Charadriiformes, such as petrels, albatrosses and shearwaters and this is under investigation.

### Future directions for identifying conserved microsatellite markers

Since this study began, four additional avian genomes have been sequenced and assembled: the turkey (*Meleagris gallopavo*), mallard duck (*Anser platyrhynchos*), collared flycatcher (*Ficedula albicollis)* and budgerigar (*Melopsittacus undulates*; as of 10th February 2013; http://www.ensembl.org/). As the costs of sequencing whole genomes continue to fall, many more bird genomes will be sequenced in the near future, so providing an increasingly rich resource for developing conserved markers. For example, following the release of the turkey and mallard genome sequence, it is now possible to identify microsatellite markers that are conserved between the chicken, turkey and mallard, and design conserved primer sets that should then amplify in a wide range of galliform and anseriform species. There are approximately 250 living species of Galliformes, which are separated from their nearest order, the Craciformes (chachalacas, curassows, guans and megapodes), by a genetic distance (Δ T_m_) of 21.6 [33]. Since the genetic distance between chicken and turkey is less than the difference between chicken and zebra finch (11.1 versus 28.0), it should be possible to create a much larger number of conserved markers for the Galliformes. However, because chicken and turkey are separated by a relatively small genetic distance (11.1), these sets would probably not be particularly highly conserved and would, therefore, be useful for only a subset of galliform species and few non–Galliformes. A comparison of zebra finch and turkey would not be expected to yield many additional new conserved microsatellite sequences, since the majority should have been identified in the zebra finch–chicken comparisons already performed (this study and Dawson *et al*. [21]). The approach used here can also be applied to the mallard genome sequence to identify highly conserved sequences and create markers (i.e. zebra finch–mallard markers) suitable for the majority of Anseriformes and Galliformes (via chicken–mallard, turkey–mallard and chicken–turkey–mallard markers).

Birds belong within the reptilian clade. Only two non-avian reptile genomes have been sequenced and assembled: the anole lizard (*Anolis carolinensis*) and Chinese softshell turtle (*Pelodiscus sinensis*) (http://www.ensembl.org/; as of 10th February 2013). The anole lizard is more closely related to birds than the turtle (http://www.ensembl.org/info/about/species_tree.pdf). Only one *CAM* locus had an identifiable lizard homologue, which included a microsatellite containing at least eight repeat units and which matched to both sides flanking the repeat region (*CAM-20*), but even for this locus it is probably not possible to create a consensus bird–lizard primer set due to low sequence similarity.

This study and that of Dawson *et al*. [21] indicate that few (if any) conserved microsatellite markers will be usefully polymorphic across *all* bird species (passerines and non-passerines). There are 23 orders of extant birds that are separated by a genetic distance (DNA: DNA melting temperature (Δ T_m_) hybridisation distance) of more than 20 [33], classification based on Sibley & Monroe [25]). This study and that of Dawson *et al*. [21] indicate that when the required (genome and/or EST) sequence data from each avian order becomes available, a conserved set of over 50 markers can be created that will be of high utility for all the species within that order. It is likely that future avian genome sequencing projects will include species originating from different bird orders and so facilitate the creation of conserved microsatellite marker sets suitable for genotyping and comparing multiple species.

## Conclusions

We have successfully developed primer sets for 24 polymorphic microsatellite loci that are of high utility in passerine birds, with some utility in non-passerine species. The microsatellite markers described here are particularly useful for genotyping species closely related to the zebra finch, such as those belonging to the Passeridae and Fringillidae families, which encompass 1,383 species [25]). When these markers are combined with 34 conserved markers developed previously [21], the requirement to isolate microsatellite loci will be alleviated for most genetic studies of passerine birds. These conserved loci are suitable for many applications, including studies of population structure, parentage and relatedness; they can also contribute towards linkage mapping and the identification of gene order rearrangements among many species. The less polymorphic loci will be useful, where required, for distinguishing between species and identifying hybrid birds (such as occur naturally in warblers, flycatchers, petrels, ducks, owls and other raptors). These loci also have potential for studying the population genetics of extinct or highly endangered species in which it is difficult to develop microsatellite libraries due to the lack of sufficient (high-quality) DNA. Conserved markers can potentially be used to genotype samples from museum collections or from other non-invasive sources (such as mouth swabs or feathers). The loci will, in particular, enable the comparison of populations and species at the same loci, and so allow genetic variability to be compared directly, without ascertainment bias.

## Abbreviations

BLAST: Basic Local Alignment Search Tool; bp: base pair; CAM: **C**onserved **A**vian **M**icrosatellite; EST: Expressed sequence tag; He: Expected heterozygosity; Ho: Observed heterozygosity; HWE: Hardy–Weinberg equilibrium; ng: nanogram; PCR: Polymerase chain reaction; RFU: Relative fluorescence unit; Ta: Annealing temperature; TG: ***T****aeniopygia guttata* – ***G****allus gallus* conserved microsatellite markers; Tm: Melting temperature

## Competing interests

The authors declare that they have no competing interests.

## Authors’ contributions

DAD coordinated the project, designed and completed the analyses and wrote the manuscript. RE and JS performed the data mining to identify numbers and frequencies of different microsatellites in the zebra finch and chicken genomes. JS identified and extracted suitable zebra finch genomic sequences and their chicken homologues from which to design the primers. ADB designed the primer sets. ADB, LGS, GJH, JP, DM-G, MM-M, AWJB, and SAJP tested primer sets in various species. LGS, IRKS and TB assisted with the analyses. ADB, LGS, DM-G, IRKS, JS and TB contributed to writing the manuscript. All authors read and approved the final manuscript.

## Supplementary Material

Additional file 1 **Genotypes observed for 12 species at 24 *CAM *** **loci.** Click here for file

Additional file 2 **Full sequence data for 24 conserved avian microsatellite (*CAM*) loci.** Click here for file

Additional file 3 **Primer melting temperatures in zebra finch and chicken for 24 conserved avian microsatellite (*CAM***) **markers.** Click here for file

Additional file 4 **Allelic richness versus genetic distance for each *CAM *** **marker.** Click here for file

Additional file 5 Typical numbers of loci polymorphic among those amplifying for a selection of passerine and non-passerine species from a selection of different studies. Click here for file

Additional file 6 **Homology of *CAM *** **loci to expressed sequence tags (ESTs), genes, other microsatellites and BACs.** Click here for file

Additional file 7 **Estimated null allele frequencies at *CAM *** **loci for six species.** Click here for file
