# Supplementary material for: High-utility conserved avian microsatellite markers enable parentage and population studies across a wide range of species
Source: BMC Genomics. 2013 Mar 15;14:176. doi: 10.1186/1471-2164-14-176 (PMC3738869; doi:10.1186/1471-2164-14-176)
Supplement: Additional file 2 — Full sequence data for 24 conserved avian microsatellite ( CAM ) loci. [file 1471-2164-14-176-S2.doc]

Dawson et al.

High-utility conserved avian microsatellite markers enable parentage and population studies across a wide range of species

**Additional file 1** Full sequence data for 24 conserved avian microsatellite (*CAM*) loci*

*Sequences exported from the zebra finch genome.

Primer bind regions and repeat regions are highlighted.

CAM-01

>2 dna:chromosome chromosome:taeGut3.2.4:2:42810182:42810619:1

ATGCTTTCTTAGCCCTTCATCAGCTACTAAACTAGTACAGGAGGAGTGCAAATCATTACTACAAAAGGCCAAGGCCAGTATGTTTTCTTAGTGAGCTTTATAGATCTTTTTTCTTATCCCTTCCTTCTTTCCCTCCTTCCCAACCACACAAAACATAACCAGCTGTAATGATAGGAGGAGAGAGCAAGCAGCTTTTCTAAACACACACACACACACACACACACACACACACACACACTCGCACTCCGGCCCCCCTCTTTCCTGCTCATACCTCAGCAGAGTGACAGCTTCTAGATGTTGCGTGCGAGTGAGTTCAGCTGGTGCTGTGTGGTCTGGGTGTGTGGTCTCCAGCTCAACCGAGAGGCTGCTAACAGGGTGGATGAGAGAGAGCAACACTGTTTACTTTAATTTATTTCGGATGCCGGAACTGTGCCCGGA

CAM-02

>7 dna:chromosome chromosome:taeGut3.2.4:7:12381541:12381972:1

GAGTGACAGATATTTCGCAGCTGATGAAATGAGAATGCAGAAATTCAAGAGTGCCTCTGGGATTTCAGTGGTCATTTGTGGCCAGCGCAAGGGCAGGGCCCGCTGCCTGGCTGGCGCAGCACTGGCTGAAGCTGCTGTTGCTGCAGGCTGCCGCAGATCCACACCATTTTAAATAATATTGGTATTATGAAGCCCTTCCCACACACACACACACACACACACACACACACACGCACTTGAGGGTATTGTGTGTGCTTTGCTTCTTTCTAGGCTGCTACTAGCTGGGAGATTGTTCTAATTGTTTTTTGTTTTGGTTTGGGGTTTTTTTTCTTCTGTCCTGTGCTCAAGCCTTCTTGAAAAGGATTTTACCTGCATCTGCTATCTTTAATTCTTTGGATAACATAAATAGAATTTACTAAATGTGATACCTAA

CAM-03

>7 dna:chromosome chromosome:taeGut3.2.4:7:9747429:9747916:1

AGTATGATGCAAACATGGGCTGATAACATCACCTTTTTTTGTTTGCCAAAGGTAAAATGTTCTTGAACTTTTTCATAGAACCAAAGAAACAAACATATAAATTAGTATCTCAGAAAAGACCACAGAAGTCTGCTGGGCACAAAACTTTTGCACCGAGCATTCAAACCTGTCATCAACAGCAGTCCGCCCAAAGAAGACCCTGTGTGTGTGTCTGTGTGTGTGTCTGTGTGTCTGTGTGTGTGTGTGTGTGTGTGTGTGTGTGTGTGTGTGTGTGTGTGTGTGTGTGTGCATTTGGCTGCTTAGACAATAAAGGACAGATTTCCTGGCACTTACAAAGAGGGGCAATGCTGAGCTATGCTAATCGGCATCATCTGGTGAGCCTACCCTGCCCAGCCACTTGAATGCAACCCCAGCAGATTCAGTGAGTTCTGTCCCCTGTGTGGGAGTCTGGTTTAAAACTGGTCACCTTGCTGCAGCCCAGATTCTGG

CAM-04

>1 dna:chromosome chromosome:taeGut3.2.4:1:34220208:34220629:1

CCAAGAGATTGGATAATATATAGGGTGTCATCATTTTAAAGCGGGTTCAGACTCAAAACTGTGGGTTTCATTTGAGTGTGACGTGAAGTATCGTATACCTCTGGCCAAGGAACTGCAGCTCATTTAATAGATAAATTGAGATGAGAAAAATGGCACAATTTAAATATCAGCTATCAACAGGAACAGGGAGGGGGAGAGATAGAGAGAGAGAGAGAGAGAGAGAAGGAGCCCAGCTTCTTTTATTACAGTCTTAGTGCTTCAACAACAGGTAGCAGTATTACATATTCCCTTTCCACTTTAAAGCTTTGGTAATTTCCTTCCTCGTGTCGGGGCGATTTTATTCATTGTTTCCTTGCAGCAGTGATTGATGTTCTGAGCTGCTAATCTCTCTGAGTGATAACTATTTTCATGTAAGTCCATGA

CAM-05

>1A dna:chromosome chromosome:taeGut3.2.4:1A:45129155:45129588:1

CCCCGCTGCCGGCTGTACTATGCGTGTGTCTTGTGTTACAGCCATGCTGCACATCGGCGGCCAGAGCGAGGGGCGCGGGGAGGAAAAGGCTGTTTACACAGACTGCAAACCGCCCGGGGAATAGTGCGGGAGAGGAAGTGAGCCGGGCCGCTGTCTGACTGCCCCGGCTTCCTGCTCGCTCCCTCTCACGGGCAGGCGCTCACACACACACACACACACACACACACACACACACCGGCCCAAGGGGAAACAAAAAAAAAAAAAGGAAAAAGGGAAAACAAAATCAGGATCTCATTACTAGAGAAACAGAGAGCCTGCAGAAGACTGTCAGCATGGAGAATCAGGGAATTTTACTCAGCTCCCTCTAGACTACCAGGAGAGAGCGGAAAAGTCCCTCAGCCGCTGGGTAGGTACAAGTGCTGCTGCGCCCGCCC

CAM-06

chromosome:taeGut3.2.4:1A:49993824:49994269:1

TATGTATATGTATATAACTAAACAGCTGCCATGTAGCAGTCACCTGGCTGGAGGGTCCTGTCCCTGCCAGCCCCCATGGCCTTTTCTTGTCACTGTAGTCTCTGGGGTGATGGTCCAGGTCTTGCCCTCAGGCAGGACTGTTGCCGTATTGCTTGCACTCTCCCACACCTCTGAGGTACGGTACCAGAATATTTATTTTTTATATATATGTATATATATATATATATTTATGTATATATATATATATTTATAAATTTTTGTTTGCCTGTTTTCTTTTTCTTGCAAATCCTTTGGAATTGCTGCCAAGGAGAAGCTAGTGAAACATGCTCCCCACGCCAGTGGCCTAATGCTCCCTCCCTGCACCGCAGGGACCCTCATCTGTTCCTCTTGCTGACTCCATCTCCCAGCTCCAGCTTGGAAGGGAGCGGGGAGCTGCGGTCCCCTGT

CAM-07

>1A dna:chromosome chromosome:taeGut3.2.4:1A:51267552:51267985:1

ATGCAGAGCCAGAGCCTCATGCTGGAGCTCCAGGAAAATGATGAGATCTGGGTGAGGCTCTACAAGCGGGAGCGGGAGAATGCCATTTACAGTGATGATGTTGATGTGTACATCACCTTCAGTGGGTACTTGGTCAAGCCCAGCATTGAATAACTCCTCTTCCTTAGGGCCTCTTAGGCCTTTTCCTTTTTCCTCTCTCCCTCTCTCTCTCTCTCTCTCTCTCTCTCTCTCTCTACTGCCCGTAGCCACTGCAAATCACTTGGAAATGGGCCTGTCAAATCTCAGGCACTGAGTGTGAAACTTGCCACACTGGCTCCCACCTCACGCTCTCTTGTAGCCAGACCTGTATCTGTCCACTTTACATCATGGCTCTACCACCTCTTGCCCCTCACCTCATAATCCCAATTTAGGTTATTGGCTTCTTCTCAATCTGC

CAM-08

>10 dna:chromosome chromosome:taeGut3.2.4:10:3390520:3390951:1

TATGCAGAATAGGTTTGAGGTTTGGCATTTTCCAGGTTCTCTGGGCCAGCTGCTGCCCACGGGGCCACCCCCAGCCCTGGTGTCAGACCGTTGGTGTTTATAGCTCGTTTCCATTGGCGTTGCTGTGTTGGGTTTCATTTCAGTCTTCCTGACTCTGCCCTTCTGTAAAGTGTACATTATTACCAAGTTCCTTGTTTTTTTATATATATATATATATAAATATATATATATACAAACTGTACTCTTCTTGCCTTTGTACATTTAGGCAAGGAGGGAGAATAAATCTTTTTAAGAGACAATCACAAACCTGTGAGGGTGGCTTTTTCTTGCTGTTCCCCCAAGTCTGGTCCTTTATCCCTAAAGAGTGTGGCCAGTTATTTTTGGGGGTGGGAGATGGGGTTTCCCCATCTCGGGCATCTGAGAAGGCTCTTG

CAM-09

>4A dna:chromosome chromosome:taeGut3.2.4:4A:8999969:9000390:1

AGTCATGGCCGTTCTAGTTTTAGAACAAAATTTCCATCTAAAAATAGTGTGGCAATCACCAGTGTAGGATCTCTGCTTTCTTAGACACACAGCCACCCCAGAGCACCTGTCATGAGAGCTGCCCCAGCACATCCCAGAGTCTGCAATATTCATGCAGAATAATGGCAATTATTCTGCATGTTGAAAATATACATTCCATCTGTGTGTGTGTGTGTGTGTGTGTCTTGGCACATGTGTATTTCCATGCTAGTACATCTGTATAATACATCTTTTCTATGTGAATTAAGCCAAACATGATCACAACATGTTATTTAAATATCACTAAGGATTAGAAGATTCAGAAAGACTCCAAACCCCATAAAGTGATGCTGAGCAGGATTACTTCCTGTCAGCATGTGGATACATGTGTAAGCTGATTATTT

CAM-10

>13 dna:chromosome chromosome:taeGut3.2.4:13:16024201:16024644:1

GGGAGCTGCATTTGTGAAGGGACCAAAAAATAAACAGCTCATTGCTCGGGTAAGTGCCAATGCGAGCTCCCGGGCTCTCATTGTCATGCTGTACATCGATCCCCCCCTCCTATTTTGTTTAGTTATACTGGGTTTGATTCTTATTAGGATTATTTGATGCATCAGCTGTATGCTTCAGAGGCTGGAGACTGACTGGTACGTGTGTGTGTGTGTGTGTGTGTGTGTGTGTGTGTGTGTGTGTGTGCGTGTGGGAAGATGCCCATTCTCTGGATATACATTGACACATTTTTCCAGCTCTCAGTGTGGTGTCTCATTCGCAGTGTTACCCCAAAGCGTGCCGTACACACACACACAAATGGTGCTTGCTGCTCCTGCCTTTAAAATCCACTCACACACGCAATTATTGAAGCTGAAAATTAAAGGGGGGGGTTCTTAAATCACAGT

CAM-11

>Z dna:chromosome chromosome:taeGut3.2.4:Z:39095963:39096408:1

GAAAGGCGGTTCAACCCAGGGGGCCAAAGGAGATGCAGGGAAAGCTTTTATTGGTATCTTCCAGCAAACGTCAGCCCAGTTAAAAAAAATCTTCGTTTCTCAGAGAGCTTTTTAACACGACTCAGGCAGCAATTGGTACAGGGACAGCAAACCAGGCTGCATCTGCTCAGCCCCAGTCACAAGCTCCCTGCTTCCTCTGAGTGTGTGTGTGTGTGTGTGTGTGTGTGTGTGTGTGTGTGTGTGTGTGCGCGCTAGACACAGGCATCCGCTCCCAGCATCTCGCTGCCTCCTGCATCCTCCGGCAGCAAGTGCAGGGCTCTGGCTTCCCGGGTACCACCCTCGCCGCTCCATCCCCTGACAGGGATTTGCACACGGGGCACGGCATGCAGTGAGCACCCGCGGGCGGAGAGGGGCTCGTGGCCAGAAGCAGCGGTGATGGACAACGT

CAM-12

>2 dna:chromosome chromosome:taeGut3.2.4:2:70094313:70094752:1

TAGTCTGACTCACCCGACAGCAAACATGTTTGCGCTCCGGCTGGTATGACTCACACTGACATTTGTCTTAAGCGTGGCAATTGTTTTAGCCCTCTGCTTGCTGCAGGATGAATGAAACGGCTGAGCTCCCCTGTTGCAGATTTACTATGTGCAGCCACCCTGGTAAATTGCAGGCTCAGCTGTGGAGTATTTCCTGTCTCACACACACACACACACACACACACACACACACACACACACGTGCATACACGTGCACAAACATACACATGCGCAGCCCCCCCCCAGTAATGCTTTACTGTTAAATGCTAGATTCATAGAAAGGAAATGCATAATTACAGCACTGCCTTTTGCACCACATAAAGCAGCCCACATCAAAAGATGTTTGTCATTAATGTTTCAACAGGTAATCTCTGGAATTACTGCCAAGCTGTGTTCCTGTT

CAM-13

>6 dna:chromosome chromosome:taeGut3.2.4:6:26899032:26899469:1

CCCATCGAGGGTTATCATTAAGTAAAGAAATAAAGAAAAAAAAACCCTGCCAGTTCCAAGAAAACCTCATCAGATAATGACCTCTGTGATTGGGTTTTCATTACCAAACAGCATCCAGAGATTGTCTGCCCCATAGAAGGAAAAAAAAAAAAAAAAAAAAAAAAAAGAAAGAAAAGAAAAAGAAAGAAAAAGCAACTGTGTCTCTCTCTCTCTTTCTCTCTCTCTCTCTCTCTCTCTCCTTTTTTTTTTGCACATCTTTTCTTTAAACCTGTCAGATCATTTCAGTATTTCAAATCCGAGGAAAACAGCCTGCCTGCTGCTGTATTTGAAGTTGTAATGGTGTCAAAAAGTCACGACTGACTGACAGCCGTCAGTCCCAGAGGAGCTCATTAAATCATAAAAACTTGACAAGGAAATAATTGAGCATCACTGGCAACT

CAM-14

>9 dna:chromosome chromosome:taeGut3.2.4:9:5386874:5387321:1

CCGCGGTTTCTGGGATAGGTGATAACTGCCAGTAATAGGATTAGCATTGATTTCTTTAGCAAGAACAGAGATGTGGCAGTTCCAGCCATTTACTGAACTGCCAGCTTTGAAATATCAAAAAAAAGAAAGAAAAAAAATTCTGCAGAAAAAAAGCTTCTTGCAGCATTTTAAAGAAAGGAAAGCCTATGTGAATTAAAATACACACACACACACACACACACACACACACACACACACACACACACACATGCACACACACACACTCACAGCCCAGCACATGGGGTATATAAATATATATATTATATGTATATTATATATATGGATCTTGCTCAGTGCTTTTTTTGTTATGGTGTGATAAAGGATCAAAAGAGCTGTCTCTGTTCTAAGCTTCGAGAAAGCCTTTTTTAAATTCATTTGGCTTTCTTTAGCTTTCACTTGCTAACATATT

CAM-15

>1A dna:chromosome chromosome:taeGut3.2.4:1A:61859551:61859976:1

TAAAGGAAATGAAAAAATGCTTCGGTTTGAGAGGACGACTCCTTTATTTCCCTTATTGGATATGTTTCATGTGTTTTCCCCAACACTTCGGCTTAAGGACATGCCTTTTGAAACACAAACACACGCACAGCTCGCACACGCACACACAGATAAGATATGTATTAGATCCTGACACATGCGGGGTGGAGGGAAGAGCGGGGGAGAGAGAGAGAGAGAGAGAGAGAGAAAGAGAAAGAGAGATCCTCCTACCTGGAGCCATAGATTGCAAGATACGTAGTGTTACCTGAGGAAGTCAGAACAAGTAAAAACAGATTGAACTTCAGGGCTCTATGAGGCAGTTGATCAAGATCAGTGCTTGCTCATTCTCTCCCTCAGACTGTCTGTCTTGGGGTTTTGTTTTACTGTTTTGAGCAGCCCTTTCCGTTT

CAM-16

>17 dna:chromosome chromosome:taeGut3.2.4:17:4368842:4369273:1

TGCACCCTAGGCTACAATTACCCCTTTATTGTCCCTTTAATGCTGTGCCAGAGAAACGTGTCTTCTGCTTTCATGTCTCTCTGATGATCTGTAATCCATACTCTGTGCAACCTGAGCAATCTATAAAGACCCAGCTCTTACTTTCAAGTGCTGCCAAAAAAAGAAATAAAACAAAATAATTACCCAGGTTTGGCGACGAGCACACACACACACACACACACACACACACACAACTATGAAATCCTGCCAAACTCTGACAGATAAAAATGTCCTCACCCTGCTTTCCTGCTGGGGTGCTGGGGAGATCTGTGTGGGGCAGGGGATGGGGTCTGTTGCTCTTTCAGTTATGGAAGGACCTCGAGGCTCTTCCCAATATCAAGGCTCTGCAGGGTGACAGGCTGGACATGAATTCAGCCAGGGAGATCCTTCCCA

CAM-17

>3 dna:chromosome chromosome:taeGut3.2.4:3:2816428:2816851:1

ACCCAATTTGCAATTTTACCATTTGTCCAGCAAGACAATGTGGCATCAGGCTGACAGCAGTAAAAGAATTTGAAAATCTCTAATTAGGAGAGCTGCGGAGCAATTAACGCATGTCAAATTTCACTTCATCAAGTGCCACTCTAATTTTCCCACTAAAAATTAATGGACTTTTTTTTTGGTGTGTGTGCCTGTGTCTCTCTGTGTGTGTGTGTGTGTGTGTGTGTCCCCTCCAGGCAATGTTGATTCAAATCTGCTCAAGATGCAGCGTTGCACAGCTAAGCATCTTCTTGATTACAACCCGACACACTTCTAACTGCTTTCATTCTCACTGCCACAGATACTTTGTCAACAGTCAAATTACGCCATGCTGATAACAATGCATAATGGTCTTCCTCAAAGGAGGCTCCTGTCATGGAGCAGCCTT

CAM-18

>3 dna:chromosome chromosome:taeGut3.2.4:3:31630754:31631186:1

TGTGGTACAGCACACTCTACAGAGAAACAGCGGAGGCCGCACAAGTCAGTTAGAGTTAAGAAGTTTACACCCAGCGCTTCTACTCAAGTCCAGTCTTAGCTTGTTTCTTATGCCCCAAGTAGCCCGCTTGTGTCCACAGCAGTCGAGTATTATCCAGTGAAGACACCAATAACATTAGCAATGTTAAAAAAAATAATCAGTATATATATATATATATATATATTATATATATATGTGTGTGTGTGTGGTTAAGATATACATATATGTATATATAGATGTGGTTAAAATATATATACATATATATATATGTATATGCATGTATACACACATATATATGTATGCATGGGAAAACCCATCAAATACACCCCTATAAGTCTTCCTGGCTCTGTTATTTAGCAGCATAAAAAGAAAAATAAAATAAAAAGTTCAAAAC

CAM-19

>1 dna:chromosome chromosome:taeGut3.2.4:1:112897782:112898213:1

GTGGCAACTCTGAGCAACACAAGCTTCATTTTGGATGGACAGACATTTCTTTCATTTAACTGTGCTTCTGATTGAGAAAACAAGATCAAGGAGGTGGTATTTGTCTTCAGCATTTATTTATCATTGGAAAAAGAACCCTTTGAGCAAGCAAAGATCACAAGCACATCACAATCAATCGGTGGTACATTAATGTGAGAGAGTGTGTGTGTGTTTGTGTGTGTGTGTGTGTGTGAGAGACTCTCATCAGAATATCAACAATGAAAAAGCACCACAAGGAGGTGTGCTTAACGTGTTTACAAGAGACAATTTGTGATATGCAACCAAACCCAGGCCACAAAATAATAATTAGACACTTCTTATCTGCCTCCAAGAGGCAAGAGTTTATGGAATGCATGGGCACCAGGTTTTGGCACCGGCTGTGCTGCAAATGAA

CAM-20

>24 dna:chromosome chromosome:taeGut3.2.4:24:5214087:5214520:1

AGTTCTCTCCTCTAGGGTAGCACCACAGTGGAGAAATCTCTTGTCTTGCAGATGAAGGATTCTCTAATAAATCTCCAGAATGGCATCGGGTCCCGGGACTTCGGGTCAGATCCAGAGGAGAAAAGGAAACGTTTCCATTAACAGGCAGGAATGCAGGAGGCCAGAGGACGGTGTGCAATATGTGTAAATAGGATATATAAATATATATATAAATATATATATATATATATATATACAGATATAAATATATATATTATATACAGATTTTAAGAGAAAAAAAAAAAAGAAAAAAAGCCTAAAGAGGGGAAGGAGCGACCTCCAACACTGGCTGAACTGGAGGGTCTCTGGGAGTGTGGGGGGGGTGTGTGTGTGGGAGGGCTGTGCTCTCCCAGCACTGAGGTGTGCTCAGGGCCGTGCCCAGCTCTGCTCCCTGG

CAM-21

>2 dna:chromosome chromosome:taeGut3.2.4:2:2028140:2028565:1

TCCGTAAACAGCTGTAAGCAATGTCTCTGGTCGGGTCAGACCGGTCTGAGTTGACAAGGCAGTGTCTCCAATTATATAGCATGCTGTAGCCTGTAAAAATGAAATCAATTTGTTATTTTTCTGGGAGAACATTATAGCGTGAGTTCACACATTTGGTGAGCCTGATGAGTCAGTGAAAGCCAGAGGAGGAACTCCAGCCCTGTGTGTGTGTGTGTGTGTGTGTGTGAGGGTCTGTGTGTGTTCAGCACAGCCCAATGCATATTCACAGCACCTTCTGCAAGGAAAGGGTGAGAAATGAGAGTAATTTGCTTTATCAGAGCAGTGGACAGTGAAAATCCTGCTGTTCTTACCTAAAACCTCAAAATGACGGCGCTCTCCGTCCGTGGTTCTCATTTCAAAATTGGCTTCATCACGGAGGAGTTTCAG

CAM-22

>18 dna:chromosome chromosome:taeGut3.2.4:18:10769784:10770211:1

AGTATTAGACATCATGATACAGAATGGTTATTCAGAGTTCACTAATTATCGCTTTGTATCTTTAAATGAGATTATAATATGGCCAGCTCTGGATCCTGGTACAGCCTGACTGAGTTATGCTGTGACACTGGGAGGCAGCAGGAAAACAGCCTGGAAAAAAAACTTCGCAAAATATTTCATATCAAGTTGGTAAAAGTCTCTGTGTGTGTGTGTGTGTGTGTGTGTGTGCAGGGCAGGAGTGAAAGTGGCCCTTCACCTCCAGGTTACAAAGACCTGTTCCAGAGCTGGGACTCCTGGCGCTCCGCTGGCTCTGCAGGTGCTCTGCAGGGGAATCTCAGCAGGCTGCACTGCACTGAACCTGCGGGGACCATCAGGGCTGGCTGCTTCCCAAACACTCCCACGCTGCTCCCATAACAAGTTGCTTTCAA

CAM-23

>6 dna:chromosome chromosome:taeGut3.2.4:6:30010998:30011433:1

AGTCCTAAGAAAAATCAAATACAACCAATTTCCATTAAAGTCCTCCTGTATTTGCTATTAAATCTAGCTTTTGACAGCATGCCACTTAATAAACATTTAATGCAGTGAGTACTTACTGCTCGGGGGTTTTAATCCCATATGATTTTCCTGTGGATTCCAAGAAGTGCCCTAGATGTCTGTCTTCTGCAAATCCAGGAGCCTGTGTGTGTGTGTGTGTGTGTGTGTGTGTGTGTGTGAGAGAGAGAGGCAGAGAGGATATATGAGTGAGAAAGAGGGCGATGTGCATTTACAAGCTAAGTGGAGATCACTGTTTTTAAACAGTCTTCACTACTAGGTCATGACAGATTTATGTATAAGCTTTTATCCCCATTGGCTGCCCTGGGTATTACGTTGCTCTTTGTGTGTTTTTTACTGATAGTAATTAGTTCTAGAATAT

CAM-24

>1A dna:chromosome chromosome:taeGut3.2.4:1A:1456627:1457052:1

GGGCCGAATCCCTATCAGTAACTCCTAAGCTCTTCAGCTGAGCTGAGTGACATCTGGAGGGGGGTTTGCCCCCCTCCATTCTCTCCCCCCCCCAACCCCCGGCTCTGGGCGAGCCAGACACGGTTTGCCCAGTGCAGAGAGAGGGGCTCCGTGTTCCGCCTCCGTGGAGTATTTGGGATTGGAGAGAGGCACACACGCGCACACACACACACACACACACACACACGCACACAGAGACCATCTGCAGTGTCAATGCGGCGCCTGCTCTGAAGACTGAAGTGGGGGGAAAGGAGAGGAGAGAAATTTAATATATATATATACATATATATATATGTATATTTACACCAAAACCCACCCAAAGCAAGCGAGGAGCATTCCCAGGTAAGGAGCCCCCCGCCCCGCCCGGGCAGCAGGGGCTGCGGGAGG
