# Supplementary material for: High-utility conserved avian microsatellite markers enable parentage and population studies across a wide range of species
Source: BMC Genomics. 2013 Mar 15;14:176. doi: 10.1186/1471-2164-14-176 (PMC3738869; doi:10.1186/1471-2164-14-176)
Supplement: Additional file 3 — Primer melting temperatures in zebra finch and chicken for 24 conserved avian microsatellite (CAM) markers. [file 1471-2164-14-176-S3.doc]

Dawson et al. High-utility conserved avian microsatellite markers enable parentage and population studies across a wide range of species

**Additional file 2** Primer melting temperatures in zebra finch and chicken for 24 conserved avian microsatellite (*CAM*) markers

| Marker | Primer sequence (5' - 3') and fluoro label ¥ | No. degen. bases in primer pair | Primer seq. homology to CH (%) (& no. of bases mis-matching)Ψ | Primer Tm (°C) and identities of bases present at the location of degenerate base(s) for ZF | Primer Tm (°C) and identities of bases present at the location of degenerate base(s) for CH | Primer Tm (°C) of remaining alternative primer states for degenerate base(s) | Diff. between F & R Tm for ZF (°C) | Diff. between F & R Tm for CH (°C) |
| --- | --- | --- | --- | --- | --- | --- | --- | --- |
| CAM-01 | [F] [HEX]AAAGGCCAAG**R**CCAGTATG | 1 | [F] 100 | [F] 60.08 (G) | [F] 55.23 (A) | [F] None | 2.76 | 2.09 |
|  | [R] CTCTCATCCACCCTGTTAGC |  | [R] 100 | [R] 57.32 | [R] 57.32 | [R] None |  |  |
|  |  |  |  |  |  |  |  |  |
| CAM-02 | [F] [6FAM]GAATTAAAGA**Y**AGCAGATGCAGG | 1 | [F] 100 | [F] 57.29 (T) | [F] 60.27 (C) | [F] None | 2.19 | 0.79 |
|  | [R] AGCTGATGAAATGAGAATGCAG |  | [R] 100 | [R] 59.48 | [R] 59.48 | [R] None |  |  |
|  |  |  |  |  |  |  |  |  |
| CAM-03 | [F] [HEX]ATTAGCATAGCTCAGCATTGCC | 1 | [F] 91 (2) | [F] 60.74 | [F] No match | [F] None | 0.09 | No match |
|  | [R] CGAGCATTCAA**M**CCTGTCATC |  | [R] 95 (1) | [R] 60.65 (A) | [R] No match | [R] 62.91 |  |  |
|  |  |  |  |  |  |  |  |  |
| CAM-04 | [F] [6FAM]TACCTCTGGC**Y**AAGGAACTG | 1 | [F] 90 (2) | [F] 60.25 (C) | [F] No match | [F] 54.71 | 1.79 | No match |
|  | [R] GCTCAGAACATCAATCACTGC |  | [R] 100 | [R] 58.46 | [R] 58.46 | [R] None |  |  |
|  |  |  |  |  |  |  |  |  |
| CAM-05 | [F] [6FAM]TTACACAGACTGCAAACCGC | 1 | [F] 100 | [F] 59.91 | [F] 59.91 | [F] None | 4.18 | No match |
|  | [R] CTGTT**K**CTCTAGTAATGAGATCCTG |  | [R] 92 (2) | [R] 55.73 (T) | [R] No match | [R] 57.72 |  |  |
|  |  |  |  |  |  |  |  |  |
| CAM-06 | [F] [HEX]GTGATGGTCCAGGTCTTGC | 0 | [F] 100 | [F] 59.04 | [F] 59.04 | [F] None | 0.31 | 0.31 |
|  | [R] CAAGAGGAACAGATGAGGGTC |  | [R] 100 | [R] 58.73 | [R] 58.73 | [R] None |  |  |
|  |  |  |  |  |  |  |  |  |
| CAM-07 | [F] [HEX]AAATGATGAG**R**TCTGGGTGAG | 2 | [F] 100 | [F] 56.61 (A) | [F] 59.00 (G) | [F] None | 0.90 | 2.47 |
|  | [R] CCATTTCCAAG**W**GATTTGC |  | [R] 100 | [R] 55.71 (T) | [R] 56.53 (A) | [R] None |  |  |
|  |  |  |  |  |  |  |  |  |
| CAM-08 | [F] [6FAM]AGAA**R**AAGCCACCCTCACAG | 1 | [F] 100 | [F] 59.33 (A) | [F] 59.45 (G) | [F] None | 3.22 | No match |
|  | [R] CTCGTTTCCATTGGCGTTG |  | [R] 95 (1) | [R] 62.55 | [R] No match | [R] None |  |  |
|  |  |  |  |  |  |  |  |  |
| CAM-09 | [F] [HEX]AGA**Y**ACACAGCCACCCCAGAG | 3 | [F] 86 (3) | [F] 64.96 (C) | [F] No match | [F] 61.48 | 7.58 | No match |
|  | [R] CAC**W**TGTATCCACA**Y**GCTGAC |  | [R] 90 (2) | [R] 57.38 (A, T) | [R] No match | [R] 56.60, 59.23, 60.05 |  |  |
|  |  |  |  |  |  |  |  |  |
| CAM-10 | [F] [6FAM]TATCC**M**GAGAATGGGCATC | 2 | [F] 89 (2) | [F] 55.94 (A) | [F] No match | [F] 60.79 | 1.89 | No match |
|  | [R] **K**GCTCTCATTGTCATGCTG |  | [R] 95 (1) | [R] 57.83 (G) | [R] No match | [R] 56.83 |  |  |
|  |  |  |  |  |  |  |  |  |
| CAM-11 | [F] [HEX]TGGTACAGGGACAGCAAACC | 1 | [F] 100 | [F] 60.95 | [F] 60.95 | [F] None | 1.41 | 3.48 |
| (Z-linked) | [R] AGATGCTG**R**GAGCGGATG |  | [R] 100 | [R] 62.36 (G) | [R] 57.47 (A) | [R] None |  |  |
|  |  |  |  |  |  |  |  |  |
| CAM-12 | [F] [6FAM]TGGCA**R**TAA**W**TCCAGAGATTACC | 3 | [F] 100 | [F] 59.51 (G,T) | [F] 59.37 (A, A), | [F] 59.37 (A, T), 59.51 (G, A) | 3.34 | No match |
|  | [R] CTG**R**CATTTGTCTTAAGCGTG |  | [R] 95 (1) | [R] 56.17 (A) | [R] No match | [R] 60.81 |  |  |
|  |  |  |  |  |  |  |  |  |
| CAM-13 | [F] [HEX]TCAAATACAGCAGCAGGCAG | 0 | [F] 100 | [F] 60.16 | [F] 60.16 | [F] None | 0.16 | 0.16 |
|  | [R] TTCATTACCAAACAGCATCCAG |  | [R] 100 | [R] 60.00 | [R] 60.00 | [R] None |  |  |
|  |  |  |  |  |  |  |  |  |
| CAM-14 | [F] [6FAM]G**Y**AAGTGAAAGCTAAAGAAAGCC | 1 | [F] 100 | [F] 60.85 (C) | [F] 55.96 (T) | [F] None | 2.69 | 2.20 |
|  | [R] GGCAGTTCCAGCCATTTAC |  | [R] 100 | [R] 58.16 | [R] 58.16 | [R] None |  |  |
|  |  |  |  |  |  |  |  |  |
| CAM-15 | [F] [6FAM]**S**GACGACTCCTTTATTTCCC | 2 | [F] 90 (2) | [F] 57.58 (G) | [F] No match | [F] 58.65 | 1.53 | No match |
|  | [R] TTCTGACTTCC**Y**CAGGTAACAC |  | [R] 100 | [R] 56.05 (T) | [R] 60.39 (C) | [R] 60.39 |  |  |
|  |  |  |  |  |  |  |  |  |
| CAM-16 | [F] [HEX]AGCCTTGAT**M**TTGGGAAGAGC | 2 | [F] 90 (2) | [F] 59.70 (A) | [F] No match | [F] 62.10 | 2.02 | No match |
|  | [R] ATCCATACTC**Y**GTGCAACCTG |  | [R] 100 | [R] 57.68 (T) | [R] 62.22 (C) | [R] 62.22 |  |  |
|  |  |  |  |  |  |  |  |  |
| CAM-17 | [F] [6FAM]CGGGTTGTAATCAAGAAGATGC | 0 | [F] 100 | [F] 60.85 | [F] 60.85 | [F] None | 0.34 | 0.34 |
|  | [R] CTGCGGAGCAATTAACGC |  | [R] 100 | [R] 60.51 | [R] 60.51 | [R] None |  |  |
|  |  |  |  |  |  |  |  |  |
| CAM-18 | [F] [HEX]TTAAGAAGTTTACACCCAGCG | 0 | [F] 100 | [F] 57.16 | [F] 57.16 | [F] None | 0.22 | 0.22 |
|  | [R] GCTAAATAACAGAGCCAGGAAG |  | [R] 100 | [R] 57.38 | [R] 57.38 | [R] None |  |  |
|  |  |  |  |  |  |  |  |  |
| CAM-19 | [F] [6FAM]TCTTGGAGGCAGATA**R**GAAGTG | 1 | [F] 100 | [F] 58.60 (A) | [F] 60.75 (G) | [F] None | 1.55 | 0.50 |
|  | [R] GAGCAAGCAAAGATCACAAGC |  | [R] 100 | [R] 60.15 | [R] 60.15 | [R] None |  |  |
|  |  |  |  |  |  |  |  |  |
| CAM-20 | [F] [HEX]TAACAGGCAGGAATGCAGG | 0 | [F] 100 | [F] 59.81 | [F] 59.81 | [F] None | 0.00 | 0.00 |
|  | [R] TCAGCCAGTGTTGGAGGTC |  | [R] 100 | [R] 59.81 | [R] 59.81 | [R] None |  |  |
|  |  |  |  |  |  |  |  |  |
| CAM-21 | [F] [6FAM]TGGGAGAACATTATAGCGTGAG | 1 | [F] 100 | [F] 59.25 | [F] 59.25 | [F] None | 0.71 | No match |
|  | [R] TTGAAATG**R**GAACCACGGAC |  | [R] 95 (1) | [R] 58.54 (A) | [R] No match | [R] 62.99 |  |  |
|  |  |  |  |  |  |  |  |  |
| CAM-22 | [F] [HEX]**R**AG**R**GCCACTTTCACTCCTG | 3 | [F] 90 (2) | [F] 61.58 (A, G) | [F] No match | [F] 57.10, 57.58, 62.14 | 2.09 | No match |
|  | [R] ATGCTGTGACACT**K**GGAGGC |  | [R] 100 | [R] 63.67 (G) | [R] 61.29 (T) | [R] None |  |  |
|  |  |  |  |  |  |  |  |  |
| CAM-23 | [F] [6FAM]CTCCACTTAGCTTGTAAATGCAC | 1 | [F] 96 (1) | [F] 58.16 | [F] No match | [F] None | 0.64 | No match |
|  | [R] CCAAG**R**AGTGCCCTAGATGTC |  | [R] 100 | [R] 58.80 (A) | [R] 61.02 (G) | [R] None |  |  |
|  |  |  |  |  |  |  |  |  |
| CAM-24 | [F] [HEX]CCCACTTCAGTCTTCAGAGC | 0 | [F] 100 | [F] 57.58 | [F] 57.58 | [F] None | 0.12 | 0.12 |
|  | [R] TGGAGTATTTGGGATTGGAG |  | [R] 100 | [R] 57.46 | [R] 57.46 | [R] None |  |  |
|  |  |  |  |  |  |  |  |  |

*, the zebra finch sequences were isolated by a search of the unassembled contigs and super contigs of the zebra finch genome and the chicken sequences were isolated by a search of the assembled chicken genome (v2.1). The sequence of each locus is provided in Supplementary Table A.;

ZF, zebra finch *Taeniopygia guttata*;

CH, chicken *Gallus gallus*;

Tm, primer melting temperature;

¥, The forward and reverse primer sequences are match 100% to zebra finch and 86–100% to chicken *Gallus gallus* when the degenerate bases are accounted for. The degenerate bases used in the primer sequences are shown in bold and underlined, R=A or G, Y= C or T, M=A or C, S=C or G, W=A or T, K=G or T;

Ψ, calculated by dividing the number of bases matching chicken (after accounting for the degenerate bases) by the total length of the primer sequence;

F, forward sequence;

R, reverse sequence;
