# Supplementary material for: High-utility conserved avian microsatellite markers enable parentage and population studies across a wide range of species
Source: BMC Genomics. 2013 Mar 15;14:176. doi: 10.1186/1471-2164-14-176 (PMC3738869; doi:10.1186/1471-2164-14-176)
Supplement: Additional file 4 — Allelic richness versus genetic distance for each CAM marker. [file 1471-2164-14-176-S4.doc]

Dawson et al.

**Additional file 4** Allelic richness versus genetic distance for each *CAM* marker
