# Supplementary material for: High-utility conserved avian microsatellite markers enable parentage and population studies across a wide range of species
Source: BMC Genomics. 2013 Mar 15;14:176. doi: 10.1186/1471-2164-14-176 (PMC3738869; doi:10.1186/1471-2164-14-176)
Supplement: Additional file 5 — Typical numbers of loci polymorphic among those amplifying for a selection of passerine and non-passerine species from a selection of different studies. [file 1471-2164-14-176-S5.doc]

**Figure 5** Number of species (a) amplified and (b) polymorphic at each individual *CAM* locus

Black bars represent passerines and grey bars non-passerines. Each locus was tested in 12 species (including zebra finch *Taeniopygia guttata* and chicken *Gallus gallus*), which included 8 passerine species, and 4 non-passerine species.

Classification of species as passerine or non-passerine was following Sibley & Monroe (1990). The data presented is based on the genotyping of 4 individuals per species.

For details of which species failed to amplify see Supplementary Table C.
