# Supplementary material for: High-utility conserved avian microsatellite markers enable parentage and population studies across a wide range of species
Source: BMC Genomics. 2013 Mar 15;14:176. doi: 10.1186/1471-2164-14-176 (PMC3738869; doi:10.1186/1471-2164-14-176)
Supplement: Additional file 6 — Homology of CAM loci to expressed sequence tags (ESTs), genes, other microsatellites and BACs. [file 1471-2164-14-176-S6.doc]

Dawson et al. High-utility conserved avian microsatellite markers enable parentage and population studies across a wide range of species

**Additional file** **6** Homology of *CAM* loci to expressed sequence tags (ESTs), genes, other microsatellites and BACs

| Marker | Homology to ESTs or genes | Details of homology to ESTs, genes, other microsatellites & BACs Ŧ |
| --- | --- | --- |
| CAM-01 | Gene | EST: None |
|  |  | GENE: HUS1 (human & mouse) |
|  |  | MSAT: None |
| CAM-02 | None | EST: None |
|  |  | GENE: None |
|  |  | MSAT: Turkey, AY552941 (CA)_13_; BAC: human AC079115 |
| CAM-03 | None | EST: None |
|  |  | GENE: None |
|  |  | MSAT: Chicken, AB075402 (CA)_2_ CG (CA)_5_ TA (CA)_6_ |
| CAM-04 | None | EST: None |
|  |  | GENE: None |
|  |  | MSAT: None |
| CAM-05 | EST & gene | EST: Chicken AJ456537; plus fat-tailed dunnart and western clawed frog |
|  |  | GENE: ELK3 (western clawed frog & chicken genome) |
|  |  | MSAT: Mallard, AY493311 (CA)_2_ GA (CA)_8_ |
| CAM-06 | Gene | EST: None |
|  |  | GENE: CACNA1I (chicken nr & chicken genome), **100545093 LOC100545093 (turkey)** |
|  |  | MSAT: None |
| CAM-07 | EST & gene | EST: Chicken CD214924; plus brown rat, four beetle, three-spined stickleback, fathead minnow |
|  |  | GENE: C1QTNF1, C1QTNF6, 100223414 LOC100223414 plus others (zebra finch, turkey, chicken, platypus, anolis lizard, zebra fish, rat), Q5ZIG9_CHICK & C1QTNF6 (chicken genome), C1QTNF6 & CSF2RB (zebra finch genome) |
|  |  | MSAT: None |
| CAM-08 | EST & gene | EST: Zebra finch CK234253; plus dog, wild boar, cow, domestic sheep, mouse, man, brown rat |
|  |  | GENE: Zfp609 (mouse, rhesus monkey, Sumatran orang-utan, chimpanzee, man, mouse, rat, chicken genome & zebra finch genome) |
|  |  | MSAT: Turkey, AY235192, (T)_4_ (TA)_8_ AA (TA)_6_ |
| CAM-09 | None | EST: None |
|  |  | GENE: None |
|  |  | MSAT: None |
| CAM-10 | EST & gene | EST: Zebra finch, FE723933 (short hit) |
|  |  | GENE: ENSTGUG00000001609 (zebra finch genome) |
|  |  | MSAT: Chicken, Z83769 (GT)_16_ |
| CAM-11 | EST & gene | EST: Chicken, CN224737 & BI391814 |
|  |  | GENE: KIAA1045 (chicken genome) |
|  |  | MSAT: Mallard AY493347 (GT)_5_ & (GT)_14_, BAC: Chicken BAC, AC192464 (GT)_11_ |
| CAM-12 | None | EST: None |
|  |  | GENE: No hits |
|  |  | MSAT: No hits |
| CAM-13 | EST & gene | EST: Zebra finch, EE050513, CK308851, DV580381, EH120076, DV582955 plus numerous animals and plants |
|  |  | GENE: VTI1A (chimpanzee, man, orangutan, white-cheeked gibbon, rhesus monkey) |
|  |  | OTHER HITS: chicken DNA (CR338821), zebra fish, sea bass, tiger puffer |
|  |  | MSAT: None |
| CAM-14 | Gene | EST: None |
|  |  | GENE: SOX14_CHICK (chicken genome, no exon within 10 Kb) |
|  |  | MSAT: Turkey, AY552870 (CA)_17_; BAC: Human AC022077, Mouse AC129193 & AC121987 |
|  |  | OTHER HITS: zebra fish (BX005459) |
| CAM-15 | Gene | EST: None |
|  |  | GENE: WNT7B_CHICK (chicken & zebra finch genome, exon located 5-10Kb distant) |
|  |  | MSAT: None |
| CAM-16 | Gene | EST: None |
|  |  | GENE: DBC1 (zebra finch genome) |
|  |  | MSAT: None |
| CAM-17 | EST | EST: Chicken, BU271161 & U266475 plus olive baboon, man, mouse |
|  |  | GENE: None |
|  |  | MSAT: Mallard AY587047 (TG)_8_CG(TG)_5_(CG)_2_(TG)_6_; turkey AF111663 (TG)_7_ CG (TG)_5_ (CG)_3_ (TG)2 CGGG (TG)_4_; BAC: Human AC007392, AK023375; Mouse cDNA AK047156, L732592 |
| CAM-18 | EST & gene | EST: Turkey EH284964, chicken BU471525 & DN852624 plus weaker hits to anolis lizard and various species |
|  |  | GENE: VEGFA (habu snake, wild boar, marmoset, white-cheeked gibbon, chimpanzee, rhesus monkey, dog, man, mouse, rat & chicken & zebra finch genome) |
|  |  | MSAT: None |
| CAM-19 | EST & gene | EST: Zebra finch EE050008, & EE051521; chicken plus numerous species |
|  |  | GENE: MAP6 (chicken, mouse & human & chicken genome) |
|  |  | MSAT: Turkey AY235040 (GT)_15_, OTHER HITS: human AP001922, AP002815, rat mRNA & BACs |
| CAM-20 | EST & gene | EST: Zebra finch EE058960; 6 hits to chicken and to numerous other species |
|  |  | GENE: 100220521 LOC100220521, 100540507 LOC100540507, 769528 GRAMD1B (zebra finch, turkey, chicken, chimpanzee, Rhesus monkey, wild boar. marmoset, dog, horse, gibbon, cow, mouse) & gene XP_002192632.1 (zebra finch genome) |
|  |  | MSAT: None |
| CAM-21 | Gene | EST: None |
|  |  | GENE: WNT3A_CHICK (chicken genome) |
|  |  | MSAT: Chicken X82793 (TG)_11_ |
| CAM-22 | None | EST: None |
|  |  | GENE: None |
|  |  | MSAT: None |
| CAM-23 | EST & gene | EST: Chicken BU255917 |
|  |  | GENE: Q5MAG4_CHICK (chicken genome), PRLHR (zebra finch genome) |
|  |  | MSAT: Grey partridge AY228558 (TG)_5_ TT (AG)_13_; Japanese quail AF121114 (TG)_7_ TT (AG)_11_ |
| CAM-24 | None | EST: None |
|  |  | GENE: None |
|  |  | MSAT: None |

Ŧ, assessed for (a) homology to sequences in the NCBI nucleotide EST and nr/nt databases identified using blastn (distant homologies) settings and (b) for homology to protein coding regions in the CH & ZF assembled genomes which was identified by the presence of exons within 5kb of the source sequence (Searches performed 30/09/2011).
